# Estimating the association between systemic Interleukin-6 and mortality in the dialysis population. Re-analysis of the global fluid study, systematic review and meta-analysis

**DOI:** 10.1186/s12882-023-03370-4

**Published:** 2023-10-26

**Authors:** Obaida Istanbuly, John Belcher, Matthew Tabinor, Ivonne Solis-Trapala, Mark Lambie, Simon J Davies

**Affiliations:** School of Medicine, Faculty of Medicine and Health Sciences, Keele University, Staffordshire, UK

**Keywords:** IL-6, Systemic inflammation, Prognosis, Hemodialysis, Peritoneal Dialysis, Mortality

## Abstract

**Background:**

Systemic inflammation, measured as circulating Interleukin-6 (IL-6) levels, is associated with cardiovascular and all-cause mortality in chronic kidney disease. However, this has not been convincingly demonstrated in a systematic review or a meta-analysis in the dialysis population. We provide such evidence, including a re-analysis of the GLOBAL Fluid Study.

**Methods:**

Mortality in the GLOBAL fluid study was re-analysed using Cox proportional hazards regression with IL-6 levels as a covariate using a continuous non-logarithmic scale. Literature searches of the association of IL-6 levels with mortality were conducted on MEDLINE, EMBASE, PyschINFO and CENTRAL. All studies were assessed for risk of bias using the QUIPS tool. To calculate a pooled effect size, studies were grouped by use of IL-6 scale and included in the meta-analysis if IL-6 was analysed as a continuous linear covariate, either per unit or per 10 pg/ml, in both unadjusted or adjusted for other patient characteristics (e.g. age, comorbidity) models. Funnel plot was used to identify potential publication bias.

**Results:**

Of 1886 citations identified from the electronic search, 60 were included in the qualitative analyses, and 12 had sufficient information to proceed to meta-analysis after full paper screening. Random effects meta-analysis of 11 articles yielded a pooled hazard ratio (HR) per pg/ml of 1.03, (95% CI 1.01, 1.03), ${I}^{2}$= 81%. When the analysis was confined to seven articles reporting a non-adjusted HR the result was similar: 1.03, per pg/ml (95% CI: 1.03, 1.06), ${I}^{2}$=92%. Most of the heterogeneity could be attributed to three of the included studies. Publication bias could not be determined due to the limited number of studies.

**Conclusion:**

This systematic review confirms the adverse association between systemic IL-6 levels and survival in people treated with dialysis. The heterogeneity that we observed may reflect differences in study case mix.

**Systematic Review Registration:**

PROSPERO - CRD42020214198.

**Supplementary Information:**

The online version contains supplementary material available at 10.1186/s12882-023-03370-4.

## Introduction

People treated with dialysis are at high risk of cardiovascular death. They have both traditional risk factors, such as smoking, hypertension, left ventricular hypertrophy, diabetes and dyslipidaemia, and non-traditional risk factors, such as protein-energy wasting, volume overload, oxidative stress, inflammation, endothelial dysfunction, uremic toxins, and hypercoagulability [1, 2]. Notably, systemic inflammation, estimated either from elevated circulating interleukin-6 (IL-6) or C-reactive protein (CRP) levels, has been reported as an important clinical factor associated with increased mortality risk, and whereas this is well documented and quantified for CRP in the general population, this is less clear for IL-6 in the dialysis CKD population. It also remains unclear whether the association between inflammation and survival in this population is causal. It is strongly associated with the risk of having cardiovascular disease and protein-energy wasting and there are potential mechanistic pathways linking these, such as the inflammatory changes associated with atheromatous plaques and activation of the ubiquitin pathway in muscle cells, but causality remains uncertain [1].

Circulating IL-6 is measured on a continuous scale which can be transformed differently when being incorporated into survival analysis. For example, concentrations are reported using different transformations including natural or base ten logarithms, or linear scales, with typical increments of either 1 or 10 pg/ml. Additionally, in many studies it is treated categorically, with divisions or cut-off values that can vary by number (e.g., quartiles or quintiles), resulting in cut-off values that are unique to the sample being analysed. Unfortunately, the estimated associations using different methods of transformation of IL-6 cannot be combined and consequently, the estimation of a pooled IL-6 effect size has proved difficult. A previous attempt to quantify the association between IL-6 and mortality risk in dialysis patients combined all these different scale approaches into a single meta-analysis [3], leading to incorrect calculation of the pooled effect size and misinterpretation of the funnel plot. Consequently, there is a need for a new systematic review and meta-analysis to estimate the association of IL-6 with mortality in dialysis patients, including re-estimation of the pooled effect size, with a view to incorporating this into future prognostic models. This information would be of value in predicting outcomes for people on dialysis and would provide an additional basis for risk stratifying of future interventions designed to reduce inflammation-related mortality.

The aim of this study was twofold: first, we re-analysed data from the Global Fluid Study (GFS), which previously reported the effect of IL-6 on survival using logarithmic (log_10_) transformation, enabling its use as a continuous scale for comparison with other studies. Second, we included this into a new systematic review and meta-analyses of the existing literature.

## Methods

### Re-analysis of the global fluid study (GFS)

The GFS design has been described previously (4), but briefly, it is an international, multi-centre, prospective longitudinal, mixed incident and prevalent observational cohort study of patients on peritoneal dialysis. Its purpose was to understand the relationships between systemic and intra-peritoneal inflammation in the context of comorbidity, dialysis prescription and peritoneal membrane function, with survival as the primary clinical outcome. Routine clinical data, including demography, comorbidity, dialysis prescription, residual and peritoneal clearances were collected electronically. In addition, samples of blood and dialysis fluid were collected, stored and analysed centrally. Baseline plasma IL-6 was measured on entry to the study (within 90 days for incident patients) and analysed using electrochemiluminescence immune assay. The single inclusion criteria was any peritoneal dialysis patient who could give informed consent. It recruited 966 patients between June 2002 and December 2008 with follow-up censored at center-specific dates in December 2010 from ten centres in the United Kingdom, Korea, and Canada. The number of all-cause mortality events was 427 during eight years follow-up. Each country obtained its ethical approval from the local ethics committees.

### Survival analysis of GFS mortality data

A Cox proportional hazards model, with stratification by centre was constructed to estimate the IL-6 effect size per pg/ml. The percentage of missing data was trivial for each variable, between 0.1 and 4%, and as the cross missing percentage was 9.4%, a complete case analysis was undertaken. Variables included in the model were age, albumin, duration of PD, and comorbidities, as previously selected in the GFS. To measure residual kidney function, renal clearance was selected instead of urine volume because this is considered a more precise measure; it also better satisfied the Cox proportional hazard assumption. Data was analysed in Stata, Version 16.

### Systematic review and meta-analyses

The systematic review of the literature was undertaken in accordance with the reporting guidelines set out by PRISMA [5], see supplementary material 5, pages 40–44, and registered with Prospero CRD42020214198 [6], where detailed description of the inclusion and exclusion criteria can be found. The following search terms were conducted in the four main health databases MEDLINE (PubMed), EMBASE (HDSA), PyschINFO (EBSCO), and CENTRAL on 04 April 2020: (“renal replacement therapy” OR “peritoneal dialysis” OR dialysis OR hemodialysis OR haemodialysis) AND (“INTERLEUKIN 6” OR “interleukin-6” OR “il-6” OR il6 OR “il 6”) AND (death OR survival OR mortality). A second search was conducted on 27 August 2021 to update with new published articles. The reference manager program, Zotero (Version 2.0.3, Virginia, US), was used to find and delete the duplications.

### Study selection criteria and data extraction

#### Inclusion criteria

- Randomized Controlled Trials, Cohort studies, including case control studies, cross sectional or longitudinal) that report mortality outcomes and/or cardiovascular outcomes in dialysis populations in which systemic inflammation, measured by plasma IL-6 levels has been measures at baseline (incident cohorts) or at entry to the study (prevalent cohorts).
- No restriction on age.

#### Exclusion criteria

- IL-6 is not included as an exposure for mortality/cardiovascular events.
- Studies less than three months duration/studies in populations in which IL-6 was measured in the context of acute illness.
- Patients undergoing transplantation only.
- Studies in vitro or in animals.
- Case reports and review articles.
- Studies not reporting mortality or cardiovascular events as an outcome.
- Studies with incomplete data or ambiguous results.
- Publications where the full text is not available.

Titles and abstracts were screened by two reviewers, OI and a research assistant. Differences were resolved by discussion with ML or SJD. For analysis of full articles a data collection form was developed by OI, ML and SJD (for details see Prospero and supplementary materials), with two reviewers (OI and MT) independently extracting data from the included studies. Discrepancies were solved by discussion. Web Plot Digitizer was used to extract the effect size values from figures when needed.

### Quality of bias assessment

Two independent reviewers (OI, MT) assessed risk of bias using the Quality of Prognostic Studies (QUIPS) tool which takes into account six separate domains: study participation, study attrition, prognostic factor measurement, outcome measurement, confounding measurement, and statistical analysis [7]. Any differences were resolved in discussion and a third reviewer (JB or SJD) was consulted. An algorithm was written for applying the QUIPS tool to avoid judgment bias between the studies, supplementary material 3, page 29–36.

#### Meta-analysis

Studies were grouped according to whether they used continuous or categorical reporting, log transformation or linear scales (either per 1 or 10 pg/ml increments). The effect size per 10 pg/ml was transformed to 1 pg/ml using the following relationship:

Hazard Ratio (HR) per 1 pg/ml = (HR per 10 pg/ml)^0.1^.

The RevMan program (Version 5.4.1) using the generic inverse variance method was used to calculate the random effect sizes in the meta-analyses [8] and heterogeneity was assessed by using ${I}^{2}$ test, to determine clinical and methodological variation [9, 10]. Sensitivity analyses were conducted to investigate the heterogeneity impact on the prognostic effect size. Publication bias was evaluated by Egger’s regression test [11].

## Results

### Global fluid study

Mean (SD) IL-6 plasma concentration for the whole cohort was 2.52 (4.86), pg/ml, median (Q1-Q3) 1.33 (0.66, 2.61), pg/ml. The combined incident and prevalent univariate HR for IL-6 for death, per pg/ml, was 1.04 (95% CI:1.03, 1.05]. The multivariable model, with adjustment for age, comorbidities (Stoke/Davies score), renal clearance, duration of PD, and albumin is shown in Table 1. Separate incident and prevalent survival models are shown in the supplementary materials 4, page 37, which show a slightly larger IL-6 effect size in the prevalent cohort. These HRs may be compared with the previous analysis of GFS in which a 215% increase in death risk was seen per log_10_ increase in IL-6 rise in incident patients, and a 268% increase per log_10_ in prevalent patients [4].

**Table 1:** Global Fluid Study: Multivariable Survival Model incorporating interleukin-6 as a continuous covariate (per 1 pg/ml)

Covariates in the Model		Unadjusted Model 1: Il-6 (per pg/ml)			Adjusted Model 2: Il-6 (per *pg/ml)*		
		HR	95% CI	p-value	**HR**	**95% CI**	**p-value**
Plasma Il-6 (per *1pg/ml)*		1.038	[1.027, 1.05]	< 0.001	1.026	[1.013, 1.039]	< 0.001
Age (per year)					1.05	[1.04, 1.06]	< 0.001
Comorbidities *(Stoke/Davies Score)*	low	Reference					
	medium				1.87	[1.45, 2.4]	< 0.001
	high				2.67	[1.92, 3.71]	< 0.001
Renal clearance (per 10 L/ week)					0.94	[0.9, 0.97]	0.001
Duration of PD (months on Dialysis)					1.01	[1, 1.02]	< 0.001
Albumin (per g/L)					0.94	[0.92, 0.96]	< 0.001
The number of participants		914			868		
The number of events		418			402		
Observations deleted due to missing data		52			98		

### Systematic review of the literature

**Fig. 1:**
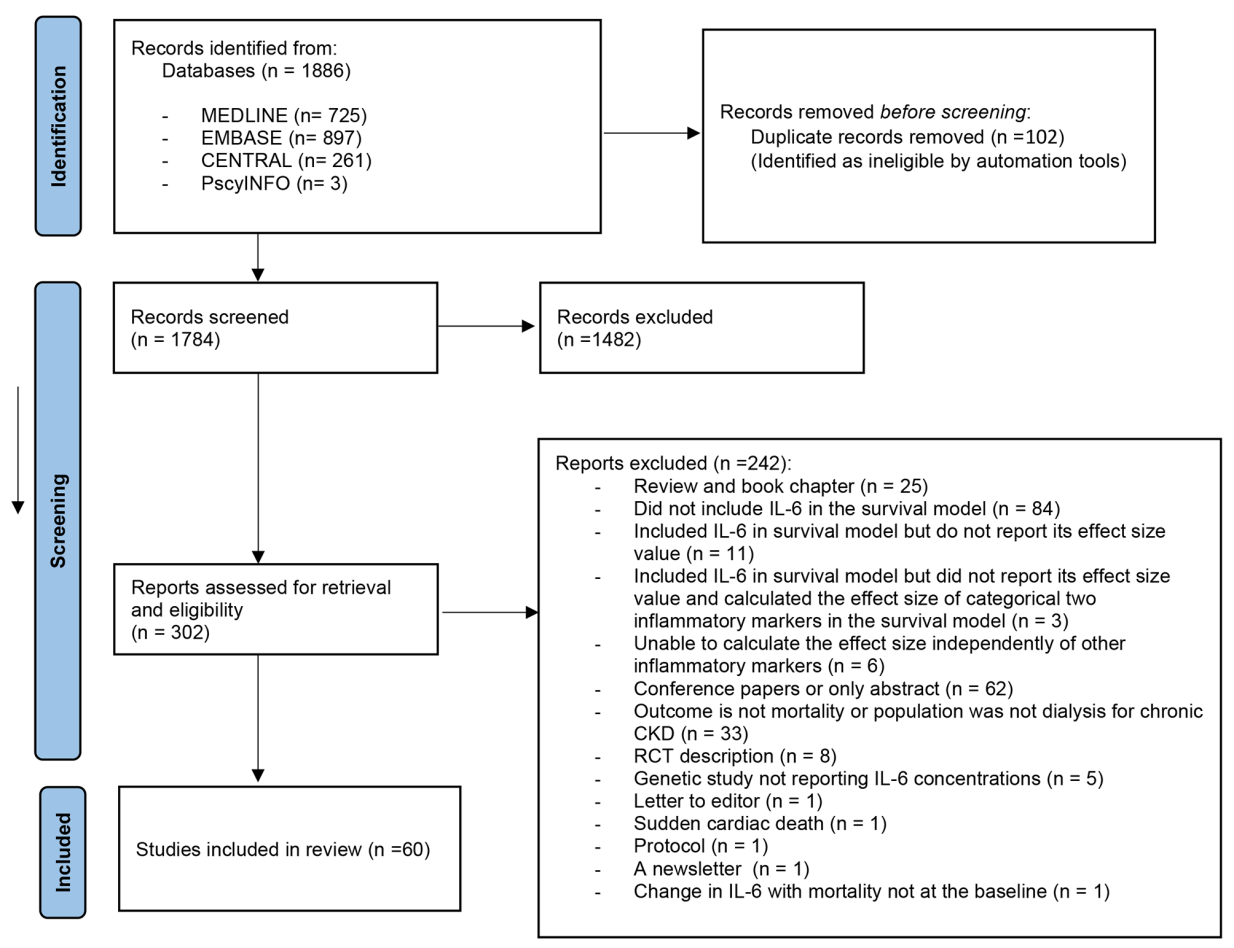
PRISMA 2020 Flow Diagram

The electronic search found 1886 citations, (see Consort Diagram, Fig. 1) of which 302 articles were eligible for full text screening, of which five were non-English language studies, (2 in German, 1 in Chinese, 1 in French, and 1 in Polish), and they were excluded after full text translation. Finally, 60 were kept, of which 12 were included in the meta-analyses [12–23].

Table 2 shows the classification of the studies according to the scale used for IL-6 in survival analyses. Thirty-six studies used IL-6 in a continuous scale, 29 (80.5%) found a significant association between IL-6 and mortality while seven (19.4%) did not; of these one (2.7%) overfitted the logistic regression model by including 10 variables with only 23 events [24], two (5.5%) had unclear modelling and regression methods [26, 27] and three (8.3%) used logarithmic transformation [28–30].

**Table 2:** Classification of the IL-6 scale found in the systematic review

Categorical only (21) *		Binary (15)
		Three categories (2)
		Four categories (3)
		Binary and four categories (1)
**Continuous only, or likely to be continuous only** (33)	Logarithmic scale (15)	Base ten (9))
		Natural Logarithm (3)
		Expressed in units of the SD (2)
		Likely to be logarithmic scale (1) (but unclear)
	Linear Scale (18)	Per 1 pg/ ml (12)
		Likely 1 pg/ ml (3) (but unclear)
		Per 10 pg/ml (3)
Both continuous and categorical* (3)		Three categories and natural logarithmic (1)
		Three categories and 1 pg/ml (1)
		Four categories and 1 pg/ml (1)
**Unclear modelling method** (3)		

In each case the number in brackets refers to the number of studies reporting using these units. In one study the units were unclear. *In total, 24 studies reported IL-6 by category

14 (58%) of 24 studies [31–44] that reported IL-6 as a categorical variable did not find a significant association with cardiovascular events or mortality or all-cause mortality in some or all of their category groups. Importantly, the cut-off was different between the studies, preventing any direct comparison, so limiting what can be concluded, (for further details see supplementary material 1, table IV, pages 8–10). The most popular method was binary categorisation (15 studies). 20 studies incorporated IL-6 as a continuous linear or presumably linear variable, 14 of them modelled it per 1 pg/ml [13–17, 19–22, 24, 25, 41, 45, 46], although for 3 of these there was uncertainty [26, 47, 48]. In addition, 3 of the 20 studies reported IL-6 as a continuous linear variable per 10 pg/ml [12, 18, 23].

Table 3 summarises those studies that reported the mean (SD) blood concentrations IL-6 in their cohort. The median of the IL-6 mean was 11.75 pg/l, and it can be noted that there is observable variation between studies, with some clear outliers. Both Kimmel 2003 [49] and Muzasti 2020 [42] included highly inflamed cohorts, which likely reflects a different case mix, although we were unable to verify this assumption. Those cohorts with particularly high levels of IL-6 tended to be of haemodialysis patients.

**Table 3:** Summary of study characteristics that reported the mean value of IL-6

First author, year (Reference)	N	Mean age, years, (SD)	Number of male (percentage)	Number of countries	Name of countries	Months on dialysis, Mean (SD)	Modality (HD or PD)	IL-6 (ng/ L)		
								Type of blood	mean	SD
Istanbuly 2023	966	55.02 (15.25)	560 (58.03)	3	the UK, Canada, Korea	8.14 (15.94)	PD	plasma	2.52	4.86
Lobo 2013 [48]	45	54.6 (14.8)	17 (37.7)	1	Brazil	62.2 (51.4)	PD	plasma	4.1	1.6
Wang 2017 [45]	177	62.4 (14.05)	112 (63.28)	1	China	35.79 (33.70)	HD	plasma	4.39	1.11
Liu 2017 [60]	50	56 (14.24)	21 (42)	1	Taiwan	Not reported	PD	plasma	5.15	6.91
Lichtenberg 2015 [19]	57	61.7 (15.9)	31 (55)	1	Israel	104.28 (71.52)	HD	serum	5.43	2.61
Han 2009 [35]	107	51.58 (11.2)	49 (45.79)	1	Korea	57.68 (19.3)	PD	serum	8.58	7.4
Panichi 2011 [34]	753	65.7 (14.2)	457 (60.7)	1	Italy	Not reported	HD	serum	8.7	14
Noori 2011 [41]	799	54 (15)	376 (47.05)	1	US	28 (26)	HD	serum	11.7	12.8
Bologa 1998 [61]	90	62	39 (43)	1	US	45.6 (not reported)	HD	plasma	11.8	Not reported
Wetmore 2008 [54]	236	62.7 (4.3)	147 (62)	1	US	64.8 (45.6)	HD	serum	13	17.5
Rao 2005 [27]	208	62.2 (12.5)	94 (45.2)	1	US	44.4 (51.6)	HD	plasma	14.9	17.5
Kalantar-Zadeh 2006 [18]	369	54.66 (14.39)	195 (54.77)	1	US	36.46 (33.81)	HD	serum	23.2	57.97
Kato 2006 [62]	154	59 (11)	101 (65.58)	Not reported	Not reported	13 (7)	HD	serum	23.16	27.86
Kalantar-Zadeh 2004 [63]	378	54.5 (14.7)	201 (53.2)	1	US	36.7 (33.9)	HD	serum	22.6	56.57
Kimmel 1998 [52]	230	54.4 (14.2)	159 (69.1)	1	US	33.7 (47.3)	HD	plasma	92.3	117.9
Kimmel 2003 [49]	240	55.1 (14.3)	175 (72.9)	1	US	49.5 (10.5)	HD	plasma	92.9	117.6
Muzasti 2020 [42]	106	53.85 (11.49)	65 (61.3)	1	Indonesia	69.43 (34.74)	HD	serum	99.66	115.87
Summary mean from all studies, 4645 patients*,									74.1	75.33

*Excluding Bologa 1998, [61] because SD was not reported and Kimmel 1998, [52] because it has 95.83% of the same population of Kimmel 2003 study [49]

Additionally, 12 studies reported IL-6 effect sizes for cardiovascular events, cardiovascular mortality, or both. It was not possible to pool them in a meta-analysis, but for a qualitative description see supplementary material Sect. 1, table II, pages 4.

### Meta-analyses of the association between systemic IL-6 and all-cause mortality

Eight studies reported a *non-adjusted* effect size of IL-6 (HR per 1 or 10 pg/ml), analysed using a continuous scale, for all-cause mortality. A pooled meta-analysis of seven of these studies, (1635 subjects), found a HR 1.03 (95% CI:1.01, 1.06; p < 0.001), *I*^2^ = 91%, *P* = 0.001. Cho 2015 [25] which reported a HR 1.07, (95%CI: 0.99, 1.15, p = 0.06) was excluded because it used a logistic regression model whereas all other studies used Cox regression. A sensitivity meta-analysis excluding the Kalantar-Zadeh 2006 study [18] because it reported a HR that was an order of magnitude different from all the other studies, yielded a similar effect size a HR 1.04 (95% CI [1.03, 1.05; p < 0.001), but with noticeably reduced heterogeneity *I*^2^ = 9%, *P* = 0.36.

**Fig. 2:**
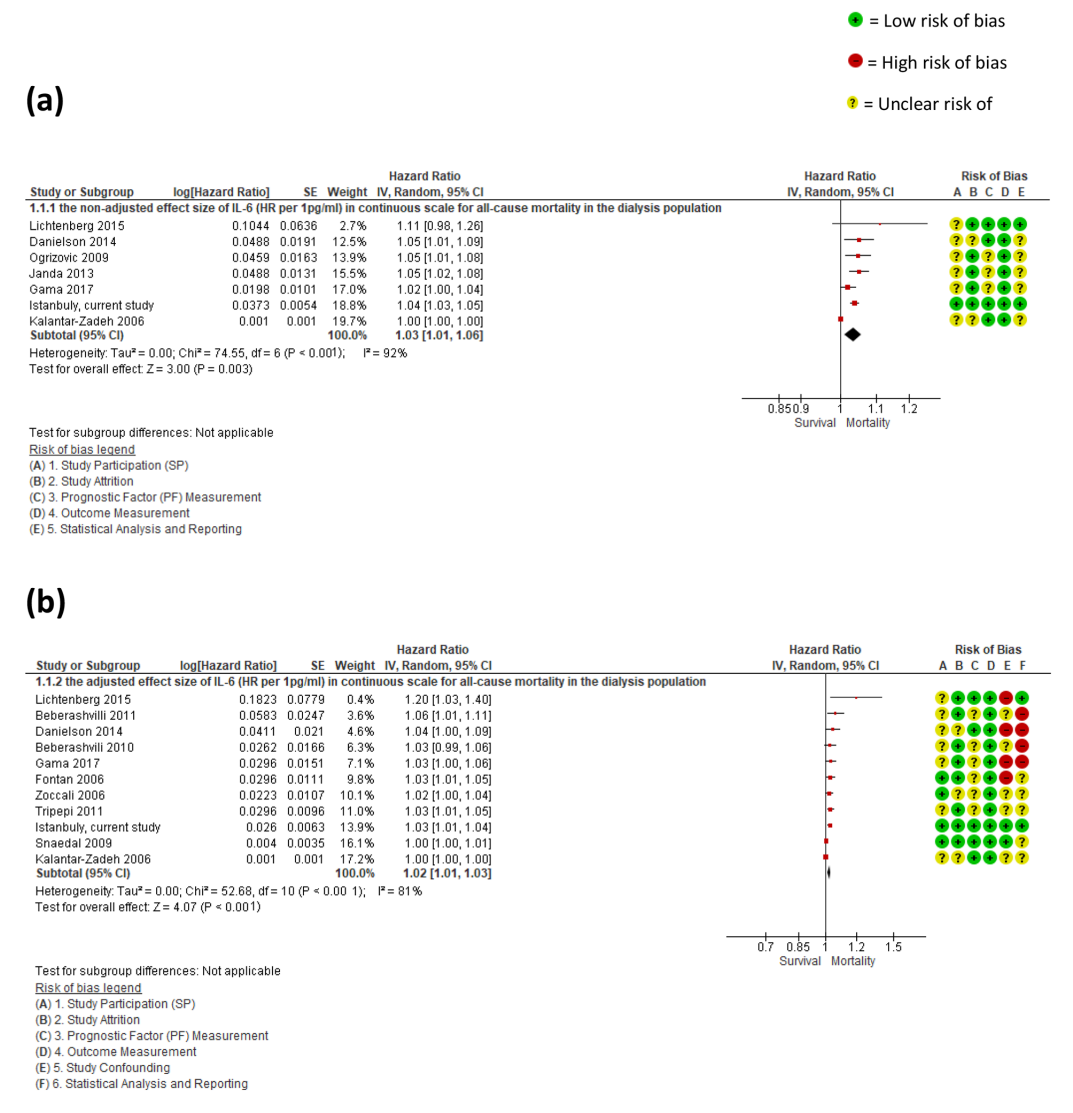
Forest plots for (**a**) the non-adjusted and (**b**) adjusted effect size of IL-6 (HR per 1pg/ml) in continuous scale for all-cause mortality in the dialysis population

The pooled meta-analysis of 11 studies that reported an *adjusted* continuous effect size for IL-6 and all-cause mortality, (2573 subjects), found a HR per pg/ml of 1.02 (95% CI: 1.01, 1.03; p < 0.001), *I*^2^ = 81%, *P* < 0.001, is shown in Fig. 2. Again, a sensitivity meta-analysis excluding two studies that reported markedly different HRs, Snaedal 2009, [21], and Kalantar-Zadeh 2006 [18] yielded a similar effect size HR of 1.03, (95% CI: 1.02, 1.04; *P* < 0.001), but a noticeably reduced heterogeneity *I*^2^ = 0%, *P* = 0.64, see supplementary material 4, page 39.

**Fig. 3:**
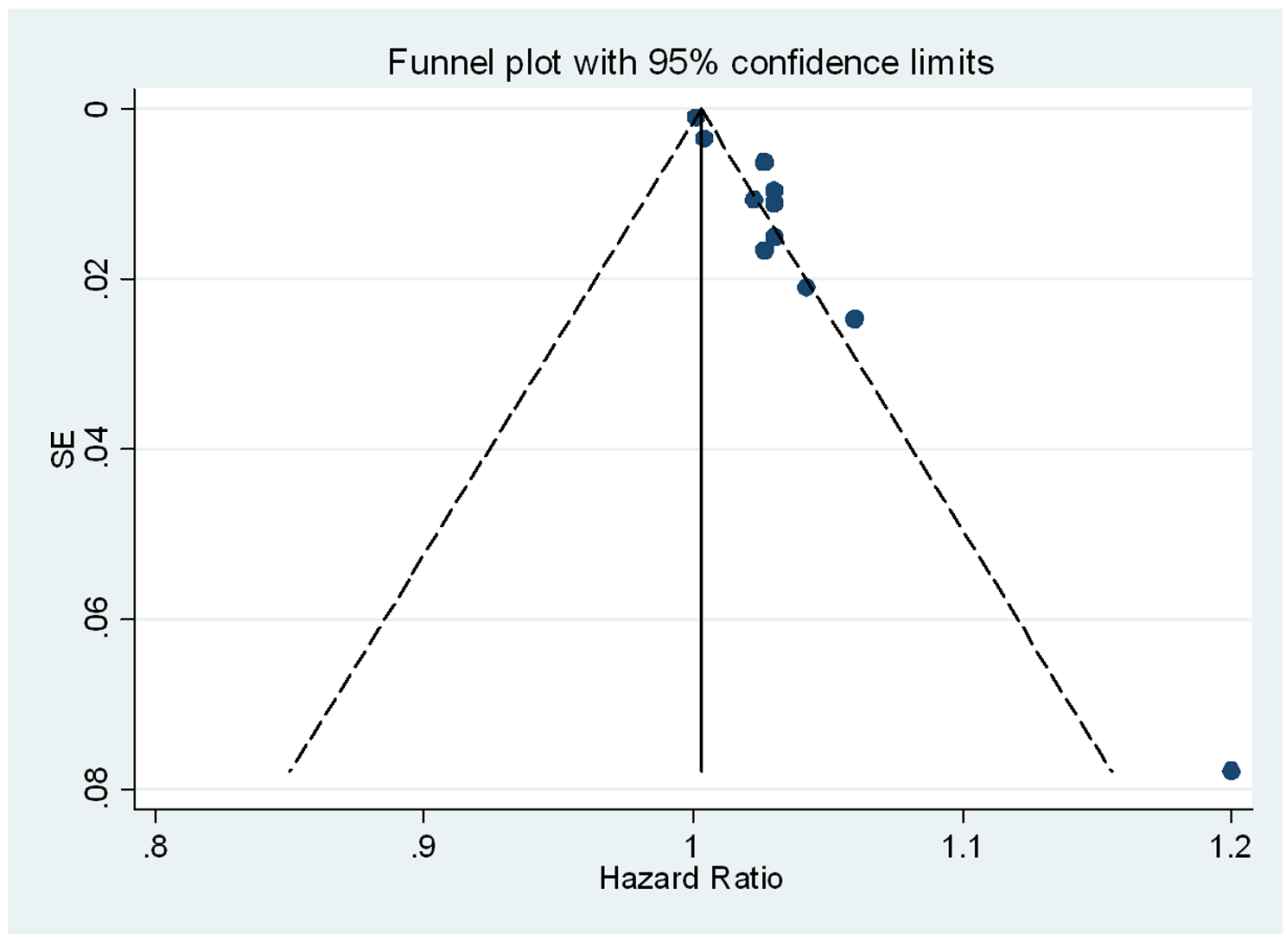
Funnel plot of the 11 included studies for effect size of IL-6 in continuous scale for all-cause mortality in dialysis population

Regarding studies reporting a logarithmic transformation of IL-6, it was not clear what base was used by Pecoits-Filho 2002 [50], Rao 2005 [27], Tsipanlis 2009 [51] or Kimmel, 2003. Holden 2013 used logistic regression model whereas other studies used Cox regression [29]. Kimmel 1998 [52], Rao 2008 [28], Lowbeer 2003 [30], and Yu 2019 [53] included the study population previously reported in new models (further details in supplementary material 1, table III, pages 5–7). Finally, Wetmore 2008 [54] and Lorenz 2018 [55] reported an adjusted effect size for IL-6 per unit of natural logarithm, analysed on a continuous scale, giving HRs of 1.41 (95% CI [1.12, 1.77]) and 1.93, 95% CI [1.4, 2.35] respectively.

### Quality assessment of risk of bias

**Fig. 4:**
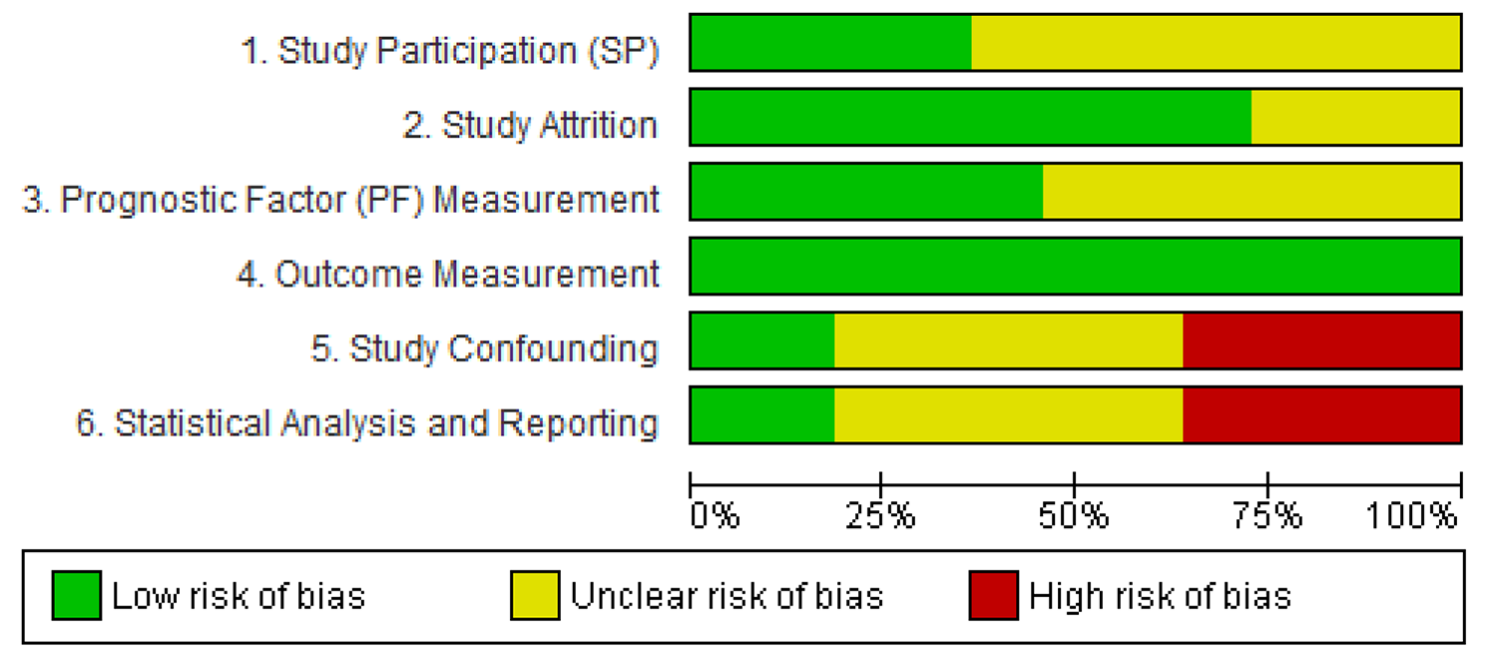
Summary of QUIPS risk of bias of studies included in the meta-analyses: each bias item is presented as percentages across all studies reporting an adjusted continuous effect size of IL-6 (HR per 1pg/ml or per 10 pg/ml)

We used the Quality in Prognosis Studies (QUIPS) tool [7, 56], see supplementary material 2 and 3, pages 14–36. There were significant concerns in the studies included in this review. Regarding potential bias in the included studies, about 60% have an unclear risk of bias in the study participation domain. Studies did not clearly report details such as sampling methods, population source, participation rate, place and date of recruitment, number and names of centres, exclusion or inclusion criteria, or the country name. In addition, some studies did not report infection status, i.e., if ‘acute infection’ was present, in their exclusion criteria which might affect the prognostic value for IL-6. In the attrition domain, about 25% had unclear risk because of not clearly reporting dropout rate and reasons for loss of follow-up. Further concerns were seen in the confounding and statistical analysis domains, with risk being unclear in more than 40% of the studies due to not reporting the proportion of missing data, imputation methods, patient numbers or the number of primary / secondary events in the model. Moreover, there were many instances where model building strategy was unclear. Additionally, more than 25% had high risk in these two domains due to overfitting the survival model, selective reporting of the results, misusing the concept of multivariate modelling or not including the key covariates age or comorbidity. For example two studies reported IL-6 in a univariable model only [17, 20]. Furthermore, Snaedal 2009 [21] and Danielson 2014 [14] did not report the value for the effect size or its 95% confidence interval. One study did not report a clear description of the type of blood sample, plasma or serum, (see Fig. 4) and Hu, 2017 [57] reported an odds ratio estimated from logistic regression, as specified in their methods, as a HR.

### Publication bias

Although this was assessed using standard methods (Fig. 3) the majority of studies which reported the adjusted and not-adjusted IL-6 effect size are located in the top of the funnel plot which means that their estimates are precise. However, the funnel plot could not confidently exclude publication bias as it was underpowered.

## Discussion

To our knowledge this is the first systematic review of the association between IL-6 and clinical outcomes in dialysis patients that considers the different methods of reporting circulating IL-6 concentrations. It confirms that IL-6 is associated with all-cause mortality in dialysis patients, supports the view that IL-6 is associated with cardiovascular mortality and demonstrates the methodological challenges of trying to combine data from different studies when reporting is not standardised. Despite this we were able to do a limited meta-analysis of studies where mortality risk was associated with IL-6 is reported linearly (either per 1 or 10 pg/ml). Of note there was considerable heterogeneity between studies with respect to their inclusion and exclusion criteria, measurement methods, quality, follow-up time, and inclusion of adjusted confounding factors. Despite this, the estimated risk associated with increasing levels of circulating IL-6 is remarkably consistent across both unadjusted and adjusted pooled estimates.

Age was the key variable included in all adjusted models, whereas comorbidities and albumin were included in half. Despite the fact that there was some inconsistency in the choice of additional variables adjusted for in the survival models the vast majority of studies reported a significant relationship between IL-6 and mortality and where meta-analyses were possible the effect size was similar (further details in the supplementary material 1, table II, page 4). However, there were some exceptions: Kalantar-Zadeh 2006 [18] and Beberashvili 2010 [12] included a large number of variables in their survival models which is a potential source of model overfitting, which can lead to erroneous conclusions. The heterogeneity observed in our meta-analyses appeared to be due to inclusion of the studies by Snaedal 2009 [21] and Kalantar-Zadeh 2006 [18]. These studies reported a log HR that was an order of magnitude smaller than for all the other studies. There was no obvious explanation for this in Snaedal’s study [21], and importantly, we could not validate their findings as they did not report mean concentration of IL-6 in the study population.

It is likely that the high degree of between study heterogeneity was due to several other factors. Case mix differed by treatment modality and there was a tendency for higher IL-6 levels in studies of HD compared to PD patients. However, there were insufficient studies with exclusively HD or PD patients to conduct a subgroup analysis to test this hypothesis. Different methods for measuring IL-6 across studies, such as the use of plasma as opposed to serum assays, may also be a source of heterogeneity. Gong et al. found that IL-6 levels were significantly higher when measured in the plasma compared with the serum [58]. Interestingly, however, our study demonstrated no such differences between plasma and serum IL-6 measures (see supplementary material 1, table I, and IV, pages 3–4 and 8–10). Whether plasma or serum Il-6 had a similar association with survival could not be tested as again there were not enough studies to undertake a subgroup analysis.

There were a number of other studies reporting the continuous effect size of IL-6 that could not be included in the meta-analyses due to uncertainties related to their reporting. Wang (2017) [45] and Prelevic (2021) reported both reported odds ratios whilst stating they were conducting Cox regressions, which would estimate hazard ratios for inference (supplementary material 1, table III, page 5 and 7). Etter (2010) was excluded because it used a logistic regression model rather than Cox regression [24]. Finally, Noori 2011 [41] was excluded because the estimate of effect could not be extracted from a graphical figure demonstrating a cubic spline Cox proportional regression estimate of IL-6 on mortality.

There are a number of limitations to this review and meta-analysis, but the main one is the variable quality of study reporting. Another limitation is the relatively low number of studies. The difference in IL-6 mean concentrations between studies was not explained and the general unexplained heterogeneity does mean that there should be caution in generalising our findings. The studies included were a mixture of incident and prevalent cohorts and there were differences in the precise timing of when IL-6 measurements were made (see supplementary material 1 Table 1). Seven studies reported IL-6 in the prevalent population, defined as being on dialysis for more than 90 days. Two studies defined the incident population as being on dialysis for less than 90 days. Two studies were not clear about IL-6 timing. It is not possible to account for all these differences in our analysis, but it is noted that in the GLOBAL Fluid Study IL-6 was an independent predictor of survival in both the incident and prevalent cohorts, with a slightly larger effect size in the prevalent cohort (see Supplementary Material, Sect. 4, page 37. Finally, while the cohort studies included in this review overwhelmingly support an association between raised circulating IL-6 levels and worse survival, cause and effect cannot be confirmed. For this, intervention studies with agents that block the activity or production of IL-6 are required. The main strength of this analysis is its attention to how the quantitative association with survival was reported and how this informed the combined in a meta-analysis using appropriate methodology.

### Recommendations and considerations for further research

**Fig. 5:**
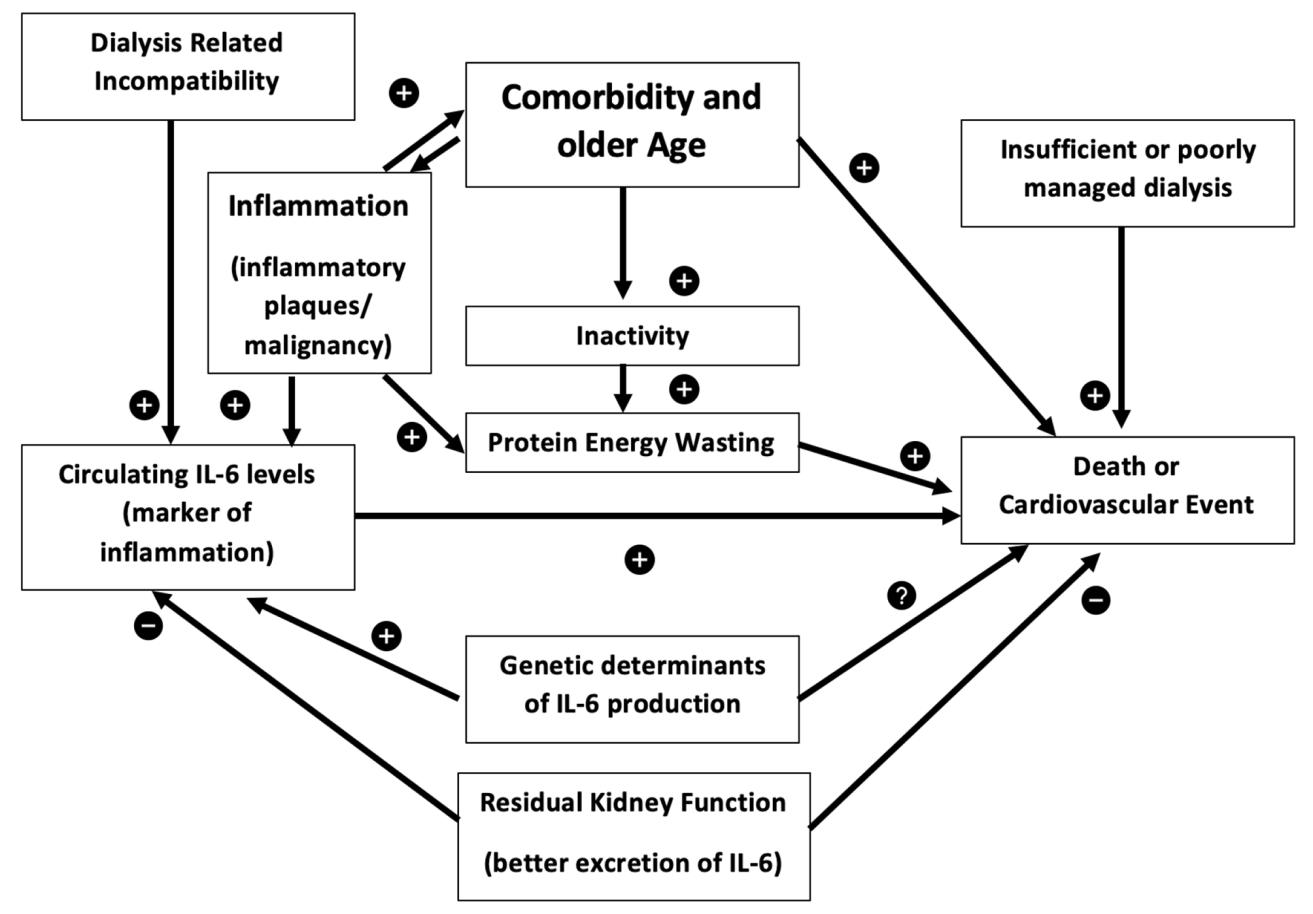
Directed acyclic graph describing the potential causal relationships between inflammation (as measured by circulating IL-6 levels) and survival/cardiovascular events in the dialysis population

Apart from general comments related to the quality of research reporting and the need to use standardized methods such as STROBE [59] and equator guidelines, perhaps the most important recommendation would be agreement on which units and scale should be used when reporting IL-6 concentrations. This analysis would suggest that categorical reporting – especially when the data is cut according to the ranges observed in a particular cohort is not helpful. Rather, using a linear reporting scale of either 1 or 10 pg/ml increments would allow comparison between groups. Given the skewed distribution of inflammatory markers in general this may seem surprising, but it does suggest that it is the lower level of inflammation that is more typical and discriminatory in dialysis patients. It would also be useful to standardize the inclusion of covariates known to be associated with mortality in adjusted survival models, taking care not to overfit models by including too many covariates. Most of the studies did not include this in their study design or analysis, but it is recommended that this should be included in the future. Although this systematic review and meta-analysis strengthens the evidence for an association between circulating levels of IL-6 and survival outcomes in dialysis patients, despite considerable heterogeneity in study design, measurement methods and survival model building strategies, it does not prove cause an effect. As indicated in Fig. 5 the relationship between comorbidity, inflammation, frailty, treatment related factors and preserved residual kidney function is complex. Further research is needed to unravel these associations, for example Mendelian randomisation studies, given that there are genetic polymorphisms that are associated with circulating IL-6 levels. These findings also support the need for randomised controlled trials of drugs targeting systematic inflammation in the dialysis population, which in addition to establishing a causal role bring the hope of improved survival in dialysis patients, whilst recognising that they are not without cost or risk.

### Electronic supplementary material

Below is the link to the electronic supplementary material.

Supplementary Material 1

## Data Availability

All data generated or analysed during this study are included in this published article [and its supplementary information files]. For the Global Fluid Study, the datasets used and/or analysed during the current study are available from the corresponding author on reasonable request.
