# Supplementary material for: Estimating the association between systemic Interleukin-6 and mortality in the dialysis population. Re-analysis of the global fluid study, systematic review and meta-analysis
Source: BMC Nephrol. 2023 Oct 26;24:312. doi: 10.1186/s12882-023-03370-4 (PMC10601265; doi:10.1186/s12882-023-03370-4)
Supplement: Supplementary file 1 — Supplementary Material 1 [file 12882_2023_3370_MOESM1_ESM.docx]

**This file consists of five groups of supplementary materials:**

- **Supplementary material (1): Characteristics of Included Studies... 3**

I. Characteristics table of the included studies in the meta-analyses (Continuous measurement) ………………………………………………………………………………………………. 3

II. Table of summary statistics and adjusted factors for the included studies in the meta-analyses (Continuous measurement) ………………………………………….………. 4

III. Summary table of statistics information of the studies which reported the association between IL-6 with outcomes per continuous change in survival model, and the reason why they have not been included in meta analyses…… 5

IV. Statistics summary table of categorical studies …..……………………………………..… 8

1. Reference of included studies…………………………………………..………………..….. 11

- **Supplementary material (2): Author judgment and support for quality assessment of the risk of bias…..………………………………..… 14**

1. Section A: Studies which reported the association between IL-6 with all-cause mortality per 1 pg/ml…………………………………………………………………………..……… 14
2. Section B: Studies which reported the association between IL-6 with all-cause mortality per 10 pg/ml ………………………………………………………………………………… 25

- **Supplementary material (3): QUIPS Tool Algorithm for Judgment the Risk of Bias for this review………………………………………….……… 29**

1. Domain 1: To judge the risk of selection bias……………………………………… 29
2. Domain 2: To judge the risk of study attrition bias……………………………. 31
3. Domain 3: To judge the risk of measurement bias of the prognostic factor (PF) ……………………………………………………………………………………………….…. 32
4. Domain 4: To judge the risk of measurement bias of the outcome……. 33
5. Domain 5: To judge the risk of measurement bias of the cofounders . 34
6. Domain 6: To judge the risk of measurement bias related to the statistical analysis and presentation……………………………………..………….. 35

- **Supplementary material (4): Statistical tables…….………….………… 37**

1. Global Fluid Study: Comparison between incident and prevalent survival models ………………………………………………………………………………………………………… 37
2. Forest plot for the non-adjusted continuous effect size of IL-6 for all-cause mortality in dialysis population after excluding Kalantar- Zadeh ……………… 38
3. Forest plot for the adjusted continuous effect size of IL-6 for all-cause mortality in dialysis population after excluding Snaedal study…………………………………….. 39

- **Supplementary material (5): PRISMA 2020 checklist CASP Checklist for systematic review ………………………………………………….………….. 40**

**Supplementary material 1: Characteristics of Studies**

It contains the five tables:

I. Characteristics table of the included studies in the meta-analyses (Continuous measurement).

II. Table of summary statistics and adjusted factors for the included studies in the meta-analyses (Continuous measurement).

III. Summary table of statistics information of the studies which calculate IL-6 effect size as a continuous change in survival model, and they have not been included in meta-analyses.

IV. Statistics summary table of categorical studies.

V. Statistics summary table of genetic studies.

# **Characteristics table of the included studies in the meta-analyses (Continuous measurement):**

| **First Author** | **Year** | **Number of countries** | **Name of countries** | **Number of**  **centers** | **Max follow up (months)** | **Exclude Infection, Yes, No,**  **Active (A)/ previous (P) / Not**  **reported**  **(- )** | **Number (N)** | **Modality** | **Incident**  **(i)/**  **Prevalent (p) / Mixed(m)** | **Time on dialysis (months)** | | **Age mean (SD)**  **(years)** | **IL-6 Measurement** | | | | | |
| --- | --- | --- | --- | --- | --- | --- | --- | --- | --- | --- | --- | --- | --- | --- | --- | --- | --- | --- |
|  |  |  |  |  |  |  |  |  |  | **Mean (SD)** | **Median Range 25^th^ to 75th** |  |  |  |  |  |  |  |
|  |  |  |  |  |  |  |  |  |  |  |  |  | **Log Transform Yes (Y) / No (N)** | **Type of log Transform (Natural (N)- Base Ten (B))** | **Plasma (P)/ Serum (S)** | **Mean, SD** | **Median, IQR** | **IL-6 Scale used in model** |
| Lichtenberg (1) | 2015 | 1 | Israel | 2 | 36 | Yes, A | 57 | HD | P | 104.28 (71.52) | N/A | 61.7 (15.9) | N | N/A | S | 5.43 (2.61) | 5.4 [0.95- 11.8] | 1 pg/ml |
| Danielson (2) | 2014 | 1 | Sweden | 2 | 36 | No | 82 | HD | P | N/A | 28 [ 7-101] | 64 (14) | N | N/A | Unclear | N/A | 6.5 [3.9–9.6] | 1 pg/ml |
| Tripepi (3) | 2011 | Unclear | Unclear | Unclear | 156 | Yes, A | 225 | HD | M | N/A | 43 [21- 109] | 60 (15) | N | N/A | S | N/A | 5 [2.7-9.2] | 1 pg/ml |
| Snaedal (4) | 2009 | 1 | Sweden | 6 | 42 | Yes. A | 228 | HD | P | N/A | 29 [14-57] | N/A | N | N/A | P | N/A | N/A | 1 pg/ml |
| Beberashvilli (5) | 2011 | 1 | Israel | 1 | 48 | Yes, A | 85 | HD | P | N/A | 33 [14.5-51.5] | 66.5 (10.6) | N | N/A | P | N/A | 6.3 [ 3.3 -11] | 1 pg/ml |
| Gama (6) | 2017 | 1 | US | 3 | 34 | No | 153 | HD | P | N/A | 28.8 [16.8 -62.4] | 60.5 (14.7) | N | N/A | Unclear | N/A | 2.1 [2.1 - 5.5] | 1 pg/ml |
| Fontan (7) | 2006 | Unclear | Unclear | Unclear | 72 | No | 133 | HD,PD | I | Unclear | Unclear | N/A | N | N/A | S | N/A | 5 [ 0-101] | 1 pg/ml |
| Janda (8) | 2013 | Unclear | Unclear | Unclear | 72 | Unclear | 55 | PD | Unclear | N/A | 24 [15- 51] | 53 (13) | N | N/A | S | N/A | 7.6 [ 3.9- 17.3] | 1 pg/ml |
| Ogrizovic (9) | 2009 | 1 | Serbia | Unclear | 36 | No | 128 | HD, PD | Unclear | 64.7 (64.8) | N/A | 53.8 (13.5) | N | N/A | S | N/A | HD 3.45 (2.87 – 4.16), PD 4.17 (3.17 – 5.48) | 1 pg/ml |
| Zoccali (10) | 2006 | 1 | Italy | 2 | 70 | Yes, A | 200 | HD | I | N/A | N/A | 61 (15) | N | N/A | S | N/A | N/A | 10 pg/ml |
| Beberashvili (11) | 2010 | 1 | Israel | 1 | 1 | Unclear | 81 | HD | P | N/A | 27 [3-183] | 64.3 (11.9) | N | N/A | P | N/A | 7.3 [0.5–152.2]* | 10 pg/ml |
| Kalantar-Zadeh (12) | 2006 | 1 | US | 8 | 36 | No | 369 | HD | P | 36.46 (33.81) | N/A | 54.66 (14.39) | N | N/A | S | 23.2 (57.97) | N/A | 1. g/L |

1. **Table of summary statistics and adjusted factors for the included studies in the meta-analyses (Continuous measurement):**

| **First Author** | **Year** | **IL-6 Modelling**  **Method** | **All-cause mortality**  **Yes(Y), No (N)** | **Cardiovascular mortality (CM)/**  **Cardiovascular Events (CE)** | **Number of Events*** | **Number of patients included in the model.** | **Name of Statistical Model** | **Number of reported multivariable models** | **Number of covariates in the model factors** | **Age** | **Comorbidity** | **Specify comorbidity** | **Albumin** | **Urine Volume or eGFR** | **BMI** | **Gender** | **Duration of PD** | **Others** |
| --- | --- | --- | --- | --- | --- | --- | --- | --- | --- | --- | --- | --- | --- | --- | --- | --- | --- | --- |
| Lichtenberg(1) | 2015 | 1 pg/ml | Y | N/A | 29 | 57 | Cox analysis | 2 | 8 | x | - | - | - | - | - | x | - | Delta IL-6 (pg/ml), Central vein catheter, Statin treatment, Previous kidney transplant Serum, Creatinine |
| Danielson (2) | 2014 | 1 pg/ml | Y | N/A | 24 | 82 | Cox Regression | 3 | 3 | x | - | - | - | - | - | x | - | - |
| Tripepi (3) | 2011 | 1 pg/ml | Y | N/A | 123 | Unclear | Cox proportional hazard ratio | 1 | 15 | x | x | Diabetes,  systolic pressure, cardiovascular (CV) comorbidities | x | - | - | x | x | smoking, cholesterol, anti- hypertensive treatment, haemoglobin, calcium phosphate, homocysteine, and ADMA |
| Snaedal (4) | 2009 | 1 pg/ml | Y | N/A | 85 | 221 | Cox Regression | 1 | 7 | x | x | Davies index | x | - | - | x | x | access type |
| Beberashvilli (5) | 2011 | 1 pg/ml | Y | N/A | 35 | Unclear | Cox proportional hazard ratio | 1 | 9 | x | x | cardiovascular disease, diabetic | x | - | - | x | x | fat mass and fat free mass |
| Gama (6) | 2017 | 1 pg /ml | Y | N/A | 29 | Unclear | Cox proportional | 1 | 7 | x | - | - | - |  | x | x | - | cause of end-stage renal disease, vascular access |
| Fontan (7) | 2006 | 1 pg/ml | Y | N/A | 42 | Unclear | Cox proportional model | 3 | Unclear | Unclear | x | Charlson score | Unclear | Unclear | Unclear | Unclear | Unclear | Unclear |
| Janda (8) | 2013 | 1 pg/ml | Y |  | Unclear | Unclear | Univariate Cox regression | 0 | 0 | - | - | - | - | - | - | - | - | - |
|  |  | 1 pg/ml |  | CM | 22 | Unclear | Univariate Cox regression | 0 | 0 | - | - | - | - | - | - | - | - | - |
| Ogrizovic (9) | 2009 | 1 pg/ml | Y | N/A | 36 | Unclear | Cox | 0 | 0 | - | - | - | - | - | - | - | - | - |
| Zoccali (10) | 2006 | 10 pg/ml | Y | N/A | Unclear | Unclear | Cox model | 1 | 10 | x | x | diabetes, previous CV events,  systolic pressure | x | - | - | x | - | haemoglobin, smoking, ADMA |
| Beberashvili (11) | 2010 | 10 pg/ml | Y | N/A | 22 | unclear | Cox proportional hazard model | 3 | 6 | x | x | diabetes and history of CV | - | - | - | x | - | phase angle, |
| Kalantar-Zadeh (12) | 2006 | 10 ng/L (pg/ml) | Y | N/A | 91 | 256 | Cox Proportional Hazard Regression | 3 | 8 | x | x | diabetes mellitus, history of cardiovascular disease, and Charlson Comorbidity Index | - | - | - | x | x | race (black) |

| *This is range value not IQR, Y yes, and N/A not applicable. |
| --- |

1. **Summary table of statistics information of the studies which reported the association between IL-6 with outcomes per continuous change in survival model, and the reasons why they have not been included in meta-analyses:**

| **First Author** | **Year** | **IL-6 Modelling**  **Method** | **All-cause mortality**  **Yes(Y), No (N)** | **Cardiovascular mortality (CM)/**  **Cardiovascular Events (CE)** | **Name of Statistical Model** | **Univariate**  **Measure** | **95 % CI** | **P Value** | **Number of reported multivariable models** | **Multivariable Measure** | | **95 % CI** | **P Value** | **Number of covariates in the model factors** | **Reason for exclusion** |
| --- | --- | --- | --- | --- | --- | --- | --- | --- | --- | --- | --- | --- | --- | --- | --- |
| Etter (13) | 2010 | Per 1 pg/ml | Y | N/A | logistic regression model | N/A | N/A | N/A | 1 | 1.049 | | [0.991, 1.111] | 0.101 | 10 | There is not enough number of papers for meta-analysis with logistic regression. Apart from the reason for exclusion, it has an overfitting model by having 10 prognostic factors for 23 events and 170 participants. |
| Noori (14) | 2011 | Per 1 pg/ml | Y | None | Cox proportional regression | Unclear | Unclear | Unclear | Unclear | Unclear | | Unclear | Unclear | Unclear | Excluded due to inability to extract effect size value from the figure which is cubic spline models with two degree of freedom of the Cox proportional regression |
| Wang (15) | 2017 | Per 1 pg/ml | Y | None | cox regression | N/A | N/A | N/A | 1 | OR 1.494 | | [1.025,2.18] | 0.37 | Unclear | Mark: The method and reported results are not clear.  Matt: Unclear in many aspects of analysis plan - final N, % of missing data and how it is dealt with, incorrect outcome ratio used for a Cox regression (should be a hazard, not odds, ratio).  Obaida: Wrong symbol for odds ratio it should be hazard ratio in cox regression model. The effect change of IL-6 is not confirmed to be 1 pg/ml. In addition, it might be inappropriate to include CRP and IL-6 in only one model as they both associated with each other and represent the same risk factor, systemic inflammation. The model might have been incorrectly specified due to adjustment of Age and Age>70 in the same model. |
| Cho (16) | 2015 | Per 1 pg/ml | Y |  | Logistic regression | OR 1.07 | [0.99, 1.15] | 0.06 | N/A | N/A | | N/A | N/A | N/A | There is not enough number of papers for meta-analysis with logistic regression. |
|  |  |  |  | CE | Poisson Regression | N/A | N/A | N/A | 1 | IRR (incidence rate ratio) 1.07 | | [1.02, 1.11] | 0.003 | 11 | There is not enough number of papers for meta-analysis for cardiovascular events. |
| Prelevic (17) | 2021 | Unclear  Maybe 1 pg/ml | Y | None | Cox regression  survival analysis | OR 0.996 95% [0.954, 1.040] P-value 0.86 | | | | | | | | | The study is poorly reported. The table head is cox regression, however, the symbol of the effect size is OR. It is not clear if they use logistic or cox regression. There is no clear description of survival model about the statistical method of il-6, whether it is pg/ml or not. However, from the description of table 1, the median reported by pg/ml, it is possible the same unit was used. In addition to that, it is not clear if the analysis is univariate or multivariate. |
| Lobo (18) | 2013 | Unclear  Maybe 1 pg/ml | N | CM | Cox proportional hazards | HR 1.44, 95% [1.03, 2.03], P-value <0.03 | | | | | | | | | There is no clear description of survival model about the scale of il-6, whether it is pg/ml or logarithmic. However, from the description of table 1, the median reported by pg/ml, it might they used the same unit. In addition to that, it is not clear if the analysis is univariate or multivariate. |
| Leal (19) | 2012 | Unclear  Maybe 1 pg/ml | N | CM | Cox proportional  hazards | N/A | N/A | N/A | 1 | 1.44 | | [1.03, 2.03] | 0.03 | Not reported | There is not enough number of papers for meta-analysis with cardiovascular mortality. In addition to that, there is no clear description of survival model about the modelling method of il-6, whether it is pg/ml or logarithmic. However, from the description of table 1, the median reported by pg/ml, it might be they use the same unit. |
| Feldreich (20) | 2019 | Unclear  Maybe logarithmic scale | N/A | CM | Cox proportional | N/A | N/A | N/A | 1 | 1.56 | | [1.14, 2.15] | 0.005 | 3 | There is not enough number of papers for meta-analysis with cardiovascular mortality. |
| Thang (21) | 2020 | Per 1 pg/ml | N | CM | Not clear  Maybe multivariate logistic regression analysis | N/A | N/A | N/A | 1 | 1.643 | | [1.38 –1.958] | < 0.001 | 6 | The study is poorly reported. The heading of the table is logistic regression, however, the symbol of the effect size is hazard ratio. It is not clear if they use logistic or cox regression. |
| Hu (22) | 2017 | Unclear  Bi | N/A | CE | logistic regression | N/A | N/A | N/A | 1 | 1.456 | | [1.078, 1.967] | 0.014 | 3 | There is not enough number of papers for meta-analysis with cardiovascular events. Apart from the reason for exclusion, it has poor reporting. The reported analysis is logistic regression, however, HR was used to report effect measure of IL-6. |
| Bi (23) | 2013 | Unclear | N/A | CE | logistic regression | N/A | N/A | N/A | 1 | 3.146 | | [2.054, 4.247] | 0.002 | 7 | There is not enough number of papers for meta-analysis with logistic regression. |
| Lambie (24) | 2013 | Based ten Logarithm | Yes | N/A | Cox regression | N/A | N/A | N/A | 1 | Incident | 2.15 | [1.22, 3.79] | 0.008 | 15 | There is not enough number of papers for meta-analysis with based ten Logarithm transform. |
|  |  |  |  |  |  |  |  |  |  | prevalent | 2.68 | [1.28, 5.61] | 0.009 | 15 |  |
| Pecoits-Filho (25) | 2002 | Based Ten logarithmic | Y | N/A | Cox proportional | N/A | N/A | N/A | 1 | 3.31 | | [1.07, 10.6] | 0.05 | 6 | There is not enough number of papers for meta-analysis with based ten Logarithm transform. |

|  |  |  |  |  |  |  |  |  |  |  |  |  |  |  |
| --- | --- | --- | --- | --- | --- | --- | --- | --- | --- | --- | --- | --- | --- | --- |
| **First Author** | **Year** | **IL-6 Modelling**  **Method** | **All-cause mortality**  **Yes(Y), No (N)** | **Cardiovascular mortality (CM)/**  **Cardiovascular Events (CE)** | **Name of Statistical Model** | **Univariate**  **Measure** | **95 % CI** | **P Value** | **Number of reported multivariable models** | **Multivariable Measure** | **95 % CI** | **P Value** | **Number of covariates in the model factors** | **Reason for exclusion** |
| Holden (26) | 2013 | Based ten Logarithm | Y | N/A | logistic regression | N/A | N/A | N/A | 1 | OR 1 | [0.9, 1.1] | 0.7 | 5 | There is not enough number of papers for meta-analysis with logistic regression. |
| Kimmel (27) | 2003 | Unclear | Y | N/A | Cox proportional hazards | N/A | N/A | N/A | 4 | 1.26 | [1.05, 1.5] | 0.01 | 10 | The logarithmic transformation was unclear. |
| Rao (28) | 2005 | Unclear | Y |  | Time dependent Cox proportional hazards | 1.24 | [1.05, 1.46] | N/A | 7 | 1.1198 | [0.951, 1.316] | N/A | 12 | The logarithmic transform was unclear. |
|  |  |  |  | Y | Cox proportional hazards | 1.44 | [1.09, 1.89] | 0.01 | 7 | 1.39 | [1.02, 1.88] | N/A | 12 |  |
| Kimmel (29) | 1998 | Based ten Logarithm | Y | N/A | Cox proportional- standardized relative risk* | N/A | N/A | N/A | 1 | 1.56 | [1.25, 1.94] | < 0.0001 | 16 | The same study population for the same author |
| Rao (30) | 2008 | Based ten Logarithm | Y |  | cox regression model | 1.24 | [1.05- 1.46] | 0.01 | 1 | 1.2 | [1.02, 1.41] | 0.03 | 6 |  |
|  |  |  | Cardiovascular hospitalization event/ all-cause mortality |  | Composite cox regression model | 1.13 | [0.98- 1.31] | 0.1 | 0 | N/A | N/A | N/A | N/A |  |
|  |  |  |  | Cardiovascular hospitalization event/ cardiovascular death | Composite cox regression model | 1.15 | [0.97- 1.36] | 0.11 | 0 | N/A | N/A | N/A | N/A |  |
|  |  |  | All-cause mortality and cardiovascular events | Time- dependent cox regression | 1.21 | [1.03, 1.41] | 0.02 | 0 | N/A | N/A | N/A | N/A | N/A |  |
| Lowbeer (31) | 2003 | Based ten Logarithm | Y | N/A | Cox proportional hazard model | N/A | N/A | N/A | 1 | 4.07 | [0.54, 30.5 ] | Not significant | 7 |  |
| Yu (32) | 2019 | Based ten Logarithm | Y | N/A | Cox proportional hazard ratio | N/A | N/A | N/A | 3 | 2.201 | [1.08, 4.47] | <0.05 | 9 |  |
| Hung (33) | 2012 | SD unit for Based ten Logarithmic Effect Size | Y |  | N/A | N/A | N/A | N/A | 1 | 1.7 | [1.1, 2.7] | 0.01 | 5 | There is not enough number papers for meta-analysis with SD unit for logarithmic base 10 scale. |
|  |  |  |  | CM | N/A | N/A | N/A | N/A | 1 | 1.6 | [1.2, 2.1] | 0.01 | 5 |  |
| Panichi (34) | 2004 | SD unit for Based ten Logarithmic Effect Size | N/A | CM | Cox Regression | N/A | N/A | N/A | 2 | 3.01 | [1.24-7.29] | Not reported | 1 |  |
| Wetmore (35) | 2008 | Natural Logarithmic | Y | N/A | Cox proportional | N/A | N/A | N/A | 2 | 1.411 | [1.124, 1.771] | 0.003 | 8 | There is not enough number of papers for meta-analysis with natural logarithmic IL-6. |
| Tsipanlis (36) | 2009 | Unclear | Y |  | Cox | 4.67 | [1.36, 16.05] | 0.014 | 0 | N/A | N/A | N/A | N/A |  |
|  |  |  |  | Y |  | 9.61 | [1.75, 52.64] | 0.009 | 0 | N/A | N/A | N/A | N/A |  |
| Lorenz (37) | 2018 | Natural Logarithm | Y |  | Cox model | N/A | N/A | N/A | 1 | 1.93 | [1.4, 2.35] | Not reported | 13 |  |
|  |  |  |  | CM |  | N/A | N/A | N/A | 1 | 1.55 | [1.1, 2.29] | Not reported | 13 |  |
| Kato (38) | 2006 | Natural logarithm |  | CM | Cox model | 6.92 | [2.07, 23.2] | <0.01 | 0 | N/A | N/A | N/A | N/A | This is only the paper which reported the effect size of logarithmic IL-6 for cardiovascular mortality. |
| Bologa(39) | 1998 | Unclear | Y |  | Cox model | 1.041 | Not reported | 0.00001 | 1 | 1.044 | Not reported | 0.001 | 4 | The scale of IL-6 in the survival model is not clear. |

* The standardized relative risk represents the increased risk associated with a 100% increase (or doubling) of the untransformed predictor variable.

1. **Statistics summary table of categorical studies:**

| **First Author** | **Year** | **All-cause mortality**  **Yes(Y), No (N)** | **Cardiovascular mortality (CM)/**  **Cardiovascular Events (CE)** | **Max Follow up**  **(months)** | **Study Sample**  **Size** | **IL-6 Measurement** | | | | | **Name of Statistical Model** | **Univariate**  **Measure** | **95 % CI** | **P Value** | **Number**  **of reported multivariable models** | **Multivariable Measure** | **95 % CI** | **P Value** | **Number of covariates in the survival model** |
| --- | --- | --- | --- | --- | --- | --- | --- | --- | --- | --- | --- | --- | --- | --- | --- | --- | --- | --- | --- |
|  |  |  |  |  |  | **Type of Categorical variable** | **Plasma (P)/ Serum (S)** | **Mean, SD** | **Median, IQR** | **Level cut-off** |  |  |  |  |  |  |  |  |  |
| Honda (40) | 2006 | Y |  | 66 | 176 | Quartiles | P | N/A | 6.4 [0.8,112] | <3.8 | Reference | | | | | | | | |
|  |  |  |  |  |  |  |  |  |  | 3.8-6.5 | Cox Hazard | HR 9.3 | [2.7-31.6] | <0.01 | 1 | HR 6.5 | [1.6, 26.6] | <0.01 | 4 |
|  |  |  |  |  |  |  |  |  |  | 6.5-10.9 |  | HR 4.2 | [1.2, 15.1] | <0.01 | 1 | HR 3.1 | [0.8,12.4] | <0.01 | 4 |
|  |  |  |  |  |  |  |  |  |  | >10.9 |  | HR 9.3 | [1.3, 17.5] | <0.01 | 1 | HR 7.7 | [1.8, 32.3] | <0.01 | 4 |
|  |  |  | Cardiovascular Events (CE) |  |  | Binary | P | N/A |  | 8.1 pg/mL | Logistic Regression | OR 4.7 | [2.4,9.2] | <0.01 | 1 | OR 2.7 | [1.1, 6.6] | < 0.05 | 8 |
| Pachaly (41) | 2008 | Y | None | 66 | 112 | Binary | P | N/A |  | 4.2 pg/mL | Cox Regression | N/A | N/A | N/A | 1 | HR 1.42 | [1.01, 2.01] | 0.03 | 7 |
| Beberashvili (42) | 2013a | Y |  | 75 | 96 | Binary | P | N/A | N/A | 6.3 pg/ml | Cox regression | HR 2.15 | [1.19, 3.86] | 0.011 | 1 | HR 2.46 | [1.28, 4.71] | 0.007 | 6 |
|  |  |  | CM |  |  |  |  | N/A | N/A |  |  | HR 2.49 | [0.93, 6.65] | 0.07 | 1 | HR 2.98 | [1.00, 8.87] | 0.05 | 6 |
| Beberasvili (43) | 2013b | Y |  | 75 | 76 | Binary | P | N/A | 6.1  [3.4, 9.7] | 6.12 pg/ml | Cox regression | HR 2.27 | [1.14, 4.53] | 0.019 | 1 | HR 2.13 | [1.02, 4.43] | 0.044 | 6 |
|  |  |  | CM |  |  |  |  |  |  |  |  | HR 2.62 | [0.89, 7.71] | 0.08 | 1 | HR 1.92 | [0.58, 6.43] | 0.29 | 6 |
| Dai  (44) | 2019 | Y | None | 60 | 358 | Binary | P | N/A | 4.2 [1.4, 14.4] | 5.1 pg/ml | $\mathrm{GENMOD}^{c}$ | RR 2.54 | [1.64, 3.93] | <0.001 | 1 | RR 1.07 | [ 0.99, 1.15] | 0.06 | 9 |
| Pa  nichi (45) | 2011 | Y |  | 36 | 753 | Binary  (IV versus I quartile) | Unclear | 8.7 (14) | IQR [3.7, 8.7] only | I: <3.7  IV: >8.7 | Cox analysis | HR 1.89 | [1.13–3.15] | 0.014 | N/A | N/A | N/A | N/A | N/A |
|  |  |  | CE |  |  |  |  |  |  |  |  | HR 0.99 | [0.98–1.01] | NS | N/A | N/A | N/A | N/A | N/A |
| Han  (46) | 2009 | N | CE | 36 | 107 | Binary | S | 8.58 (7.4) | N/A | 7.0 pg/ml | Cox regression | N/A | N/A | N/A | 1 | HR 1.52 | [0.32,13.6] | NS | 4 |
| Wang (47) | 2009 | Y |  | 48 | 231 | Binary | S | N/A | 5.20 [9.70, 17.80] | 14.07 pg/mL | Cox regression | HR 2.47 | [1.52, 4.00] | P <  0.001 | N/A | N/A | N/A | N/A | N/A |
|  |  |  | CM |  |  |  |  |  |  |  |  | HR 2.33 | [1.53, 3.55] | P <  0.001 | N/A | N/A | N/A | N/A | N/A |
| Beberashvili (48) | 2013 | Y |  | 48 | 94 | Binary | P | N/A | N/A | 6.4 pg/mL | Cox regression | N/A | N/A | N/A | 1 | HR 2.48 | [1.20, 5.13] | 0.014 | 4 |
|  |  |  | CM |  |  |  |  |  |  |  |  | N/A | N/A | N/A | 1 | HR 2.73 | [0.86,8.71] | 0.089 | 4 |
| Carrero (49) | 2008W | Y | N/A | 42 | 175 | Binary | P | N/A | 8.7 | 7.0 pg/ mL | Cox proportional | N/A | N/A | N/A | 2 | HR 2.51 | [1.37,4.75] | 0.002 | 5 |
| Hung (50) | 2005 | Y | N/A | 36 | 158 | Binary | S | N/A | N/A | 7.26 pg/ mL | Logistic regression | N/A | N/A | N/A | 2 | OR 2.23 | [0.58, 8.6] | 0.245 | 12 |
| Hasuike (51) | 2009 | Y | N/A | 87 | 120 | Binary | P | N/A | 4.47 [1.80, 9.12] | 4.5 pg/ml | Cox proportional | HR 6.375 | [1.426,28.491] | P < 0.05* | N/A | N/A | N/A | N/A | N/A |
| Liu (52) | 2017 | Y | N/A | 84 | 50 | Binary | P | 5.15 (6.91) | N/A | 3.92 pg/ml | Cox regression model | OR 6.9 | Not Reported | <0.001 | N/A | N/A | N/A | N/A | N/A |
| Sun (53) | 2016 | Y |  | 60 | 543 | Binary | P | N/A | N/A | 6.7 pg/ml | Logistic regression | N/A | N/A | N/A | 2 | HR 1.79 | [1.20,2.67] | 0.01 | 22 |
|  |  |  | CM |  |  |  |  |  |  |  | Logistic regression | N/A | N/A | N/A | 1 | HR 1.43 | [0.83,2.48] | 0.2 | 22 |
|  |  |  | CE |  |  |  |  |  |  | 7 pg/ml | generalized linear model (GENMOD) Regression | N/A | N/A | N/A | 1 | RR 1.10 | [1.02, 1.19] | 0.01 | 18 |

| **First Author** | **Year** | **All-cause mortality**  **Yes(Y), No (N)** | **Cardiovascular mortality (CM)/**  **Cardiovascular Events (CE)** | **Max Follow up**  **(months)** | **Study Sample**  **Size** | **IL-6 Measurement** | | | | | **Name of Statistical Model** | **Univariate**  **Measure** | **95 % CI** | **P Value** | **Number**  **of reported multivariable models** | **Multivariable Measure** | **95 % CI** | **P Value** | **Number of covariates in the survival model** |
| --- | --- | --- | --- | --- | --- | --- | --- | --- | --- | --- | --- | --- | --- | --- | --- | --- | --- | --- | --- |
|  |  |  |  |  |  | **Type of Categorical variable (Binary/ Tertiles /Quartiles )** | **Plasma (P)/ Serum (S)** | **Mean, SD** | **Median, IQR** | **Level cut-off** |  |  |  |  |  |  |  |  |  |
| Cho (16) | 2015 | Y |  | 24 | 170 | Tertile | S | N/A | 1.56 [1.05 - 2.99] | Unclear  The highest tertile | Logistic regression | OR 10.72 | [1.31, 87.75] | 0.03 | N/A | N/A | N/A | N/A | N/A |
| Panichi (54) | 2008 | Y |  | 30 | 757 | TertileW | S | N/A | 8.6 [2.8, 6.9] | 1st <3.2, | Cox regression analysis | Reference | | | N/A | N/A | N/A | N/A | N/A |
|  |  |  |  |  |  |  |  |  |  | 2^nd^ 3.2-5.8, |  | 1.27 | [0.81, 1.98] | NS |  |  |  |  |  |
|  |  |  |  |  |  |  |  |  |  | 3rd > 5.8 pg/ml |  | RR 1.74 | [1.15, 2.61] | 0.006 |  |  |  |  |  |
|  |  |  | CM |  |  |  |  |  |  | 1st <3.2, |  | Reference | | | N/A | N/A | N/A | N/A | N/A |
|  |  |  |  |  |  |  |  |  |  | 2^nd^ 3.2-5.8, |  | RR 1.23 | [0.62, 2.42] | NS |  |  |  |  |  |
|  |  |  |  |  |  |  |  |  |  | 3rd > 5.8 pg/ml |  | RR 1.46 | [0.77, 2.77] | NS |  |  |  |  |  |
|  |  |  | Non-fatal CE |  |  |  |  |  |  | 1st <3.2, |  | Reference | | | N/A | N/A | N/A | N/A | N/A |
|  |  |  |  |  |  |  |  |  |  | 2^nd^ 3.W2-5.8, |  | RR 1.40 | [0.76, 2.56] | NS |  |  |  |  |  |
|  |  |  |  |  |  |  |  |  |  | 3rd > 5.8 pg/ml |  | RR 1.58 | [0.88, 2.83] | NS |  |  |  |  |  |
| Tripepi (55) | 2005 | Y |  | 70 | 217 | Tertile | S | N/A | 6.1 [3.0, 10.3] | 1st <3, | Cox regression analysis | Not reported | | | 1 | Reference | | | 8 |
|  |  |  |  |  |  |  |  |  |  | 2^nd^ 3- 10.3, |  |  |  |  |  | Not reported | | |  |
|  |  |  |  |  |  |  |  |  |  | 3rd > 10.3 pg/ml |  |  |  |  |  | HR 2.09 | [1.01, 3.94] | N/A |  |
|  |  |  | CM |  |  |  |  |  |  | 1st <3, | Cox regression analysis | Not reported | | | 1 | Reference | | | 8 |
|  |  |  |  |  |  |  |  |  |  | 2^nd^ 3- 10.3, |  |  |  |  |  | Not reported | | |  |
|  |  |  |  |  |  |  |  |  |  | 3rd > 10.3 pg/ml |  |  |  |  |  | HR 2.51 | [1.06, 4.93] | N/A |  |
| Panichi  (34) | 2004 |  | CM | 48 | 162 | Quartiles | P | N/A | 6.2 [1.2, 51.2] | 1st <3.6 | Cox regression | Reference | | | 3 | Reference | | | 12 |
|  |  |  |  |  |  |  |  |  |  | 2^nd^ 3.6–6.2, |  | RR 0.84 | [0.28, 2.49] |  |  | RR 1.2 | [0.34,4.28] | Not reported |  |
|  |  |  |  |  |  |  |  |  |  | 3^rd^ 6.3–13.9 |  | RR 1.83 | [0.68, 4.95] |  |  | RR 1.41 | [0.40,4.92] | Not reported |  |
|  |  |  |  |  |  |  |  |  |  | 4^th^ >13.9 pg/ml |  | RR 5.2 | [2.06, 13.11] |  |  | RR 4.26 | [1.12,16.19] | Not reported |  |
| Noori  (14) | 2011 | Y |  | 75 | 279 African Americans | Quartiles | S | 11.7  (12.8) | N/A | Q 1 (The value is not reported) | Cox regression | N/A | N/A | N/A | 1 | Reference | | | 5 |
|  |  |  |  |  |  |  |  |  |  | Q 2 (The value is not reported) |  | N/A | N/A | N/A |  | HR 1.04 | [0.57, 1.89] | Not significant |  |
|  |  |  |  |  |  |  |  |  |  | Q 3 (The value is not reported) |  | N/A | N/A | N/A |  | HR 1.53 | [0.87, 2.71] | Not significant |  |
|  |  |  |  |  |  |  |  |  |  | Q 4 (The value is not reported) |  | N/A | N/A | N/A |  | HR 2.2 | [1.28, 3.79] | <0.05 |  |
|  |  |  |  |  | 520 All Whites |  |  | 12.2 (14.84) |  | Q 1 (The value is not reported) |  | N/A | N/A | N/A | 1 | Reference | | | 5 |
|  |  |  |  |  |  |  |  |  |  | Q 2 (The value is not reported) |  | N/A | N/A | N/A |  | HR 1.88 | [0.97, 3.66] | Not significant |  |
|  |  |  |  |  |  |  |  |  |  | Q 3 (The value is not reported) |  | N/A | N/A | N/A |  | HR 2.29 | [1.2,4.37] | <0.05 |  |
|  |  |  |  |  |  |  |  |  |  | Q 4 (The value is not reported) |  | N/A | N/A | N/A |  | HR 4.13 | [2.22, 7.67] | <0.05 |  |
| Badiou (56) | 2008 | Y |  | 51 | 134 | Textile | P | N/A | N/A | <6.975 | Cox proportional  hazards regression | N/A | N/A | N/A | 1 | Reference | | | 4 |
|  |  |  |  |  |  |  |  |  |  | 6.975 to 16.618 |  | N/A | N/A | N/A |  | HR 2.90 | [1.22, 6.89] | 0.02 |  |
|  |  |  |  |  |  |  |  |  |  | ≥ 16.619 ng/ml |  | N/A | N/A | N/A |  | HR 4.61 | [1.91, 11.16] | 0.001 |  |
|  |  |  | CM |  |  |  |  |  |  | <6.975 |  | N/A | N/A | N/A | 1 | Reference | | | 4 |
|  |  |  |  |  |  |  |  |  |  | 6.975 to 16.618 |  | N/A | N/A | N/A |  | HR 7.03 | [1.59, 31.10] | 0.01 |  |
|  |  |  |  |  |  |  |  |  |  | ≥ 16.619 ng/ml |  | N/A | N/A | N/A |  | HR 11.32 | [2.52, 50.90] | 0.002 |  |
| Kalantar-Zadeh (57) | 2004 | Y |  | 12 | 378 | Quartiles | S | 22.6 (56.57) | 8.65 [4.96, 17.91] | Fourth vs  the first quartile (pg/ml) | Cox  proportional hazard models | N/A | N/A | N/A | 1 | HR 27.44 | [3.52, 213.74] | 0.002 | 11 |
|  |  |  |  |  |  |  |  |  |  | Fourth quartile vs the rest |  | N/A | N/A | N/A | 1 | HR 3.97 | [2.02, 7.79] | <0.001 |  |
| Muzasti (58) | 2020 | Y |  | 9 | 106 | Binary | S | 99.66 (115.87) | N/A | 86.9 pg/ml | Cox regression | HR 3.64 | [1.16, 11.48] | 0.03 | 3 | HR 4.15 | [0.83, 20,77] | 0.08 | 4 |
| Gergei (59) | 2020 | Y |  | 138.48 | 41 | Binary | P | N/A | 3.2 [2.2, 5.2] | 3.17pg/ml | Cox proportional hazard regression | HR 1.73 | [0.82, 3.65] | 0.15 | 0 | N/A | N/A | N/A | N/A |
| Meuwese (60) | 2011 | Y |  | 42 | 201 | Quartiles | P | N/A | 8.6 [5.1–15.0] | Stable low | Cox proportion  al hazards  model | Reference | | | 1 | Reference | | | |
|  |  |  |  |  |  |  |  |  |  | Decrease |  | HR 1.94 | [1.02, 3.68] | N/A |  | HR 1.76 | [0.92, 3.38] | N/A | 5 |
|  |  |  |  |  |  |  |  |  |  | Increase |  | HR 3.38 | [1.86, 6.14] | N/A |  | HR 3.62 | [1.96, 6.69] | N/A |  |
|  |  |  |  |  |  |  |  |  |  | Stable high |  | HR 5.73 | [3.3, 9.97] | N/A |  | HR 3.8 | [2.1, 6.87] | N/A |  |

**A Decreased IGF-1 levels potentiate association of inflammation with all-cause and cardiovascular mortality in prevalent hemodialysis patients,** [**http://dx.doi.org/10.1016/j.ghir.2013.07.005**](http://dx.doi.org/10.1016/j.ghir.2013.07.005)**.**

**B Increased basal nitric oxide amplifies the association of inflammation with all-cause and cardiovascular mortality in prevalent hemodialysis patients, DOI 10.1007/s11255-013-0436-9.**

**C Generalized linear regression models in SAS.**

**E they add CRP and IL-6 in the same model which might cause collinearity issue**

**F it reported as a serum in the method and plasma in the results.**

**G. The trajectory status was classified depend on three months period follow-up**

Reference of included studies:

1. Lichtenberg S, Korzets A, Zingerman B, Green H, Erman A, Gafter U, et al. An Intradialytic Increase in Serum Interleukin-6 Levels is Associated with an Increased Mortality in Hemodialysis Patients. Int J Artif Organs. 2015 May 1;38(5):237–43.

2. Danielson K, Beshara S, Qureshi AR, Heimbürger O, Lindholm B, Hansson M, et al. Delta-He: a novel marker of inflammation predicting mortality and ESA response in peritoneal dialysis patients. Clin Kidney J. 2014 Jun;7(3):275–81.

3. Tripepi G, Mattace Raso F, Sijbrands E, Seck MS, Maas R, Boger R, et al. Inflammation and asymmetric dimethylarginine for predicting death and cardiovascular events in ESRD patients. Clin J Am Soc Nephrol CJASN. 2011 Jul;6(7):1714–21.

4. Snaedal S, Heimbürger O, Qureshi AR, Danielsson A, Wikström B, Fellström B, et al. Comorbidity and acute clinical events as determinants of C-reactive protein variation in hemodialysis patients: implications for patient survival. Am J Kidney Dis. 2009;53(6):1024–33.

5. Beberashvili I, Sinuani I, Azar A, Yasur H, Shapiro G, Feldman L, et al. IL-6 Levels, Nutritional Status, and Mortality in Prevalent Hemodialysis Patients. Clin J Am Soc Nephrol. 2011 Sep 1;6(9):2253.

6. Flores Gama C, Rosales LM, Ouellet G, Dou Y, Thijssen S, Usvyat L, et al. Plasma Gelsolin and Its Association with Mortality and Hospitalization in Chronic Hemodialysis Patients. Blood Purif. 2017;43(1–3):210–7.

7. Fontán MP, Máñez R, Rodríguez-Carmona A, Peteiro J, Martínez V, García-Falcón T, et al. Serum levels of anti-alphaGalactosyl antibodies predict survival and peritoneal dialysis-related enteric peritonitis rates in patients undergoing renal replacement therapy. Am J Kidney Dis Off J Natl Kidney Found. 2006 Dec;48(6):972–82.

8. Janda K, Krzanowski M, Dumnicka P, Kuśnierz-Cabala B, Sorysz D, Sułowicz W. Hepatocyte growth factor as a long‑term predictor for total and cardiovascular mortality in patients on peritoneal dialysis. Pol Arch Med Wewn. 2013;123(9):453–9.

9. Simic Ogrizovic S, Jovanovic D, Dopsaj V, Radovic M, Sumarac Z, Bogavac SN, et al. Could depression be a new branch of MIA syndrome? Clin Nephrol. 2009 Feb;71(2):164–72.

10. Zoccali C, Mallamaci F, Tripepi G, Cutrupi S, Pizzini P. Low triiodothyronine and survival in end-stage renal disease. Kidney Int. 2006 Aug;70(3):523–8.

11. Beberashvili I, Azar A, Sinuani I, Yasur H, Feldman L, Averbukh Z, et al. Objective Score of Nutrition on Dialysis (OSND) as an alternative for the malnutrition-inflammation score in assessment of nutritional risk of haemodialysis patients. Nephrol Dial Transplant Off Publ Eur Dial Transpl Assoc - Eur Ren Assoc. 2010 Aug;25(8):2662–71.

12. Kalantar-Zadeh K, Brennan ML, Hazen SL. Serum myeloperoxidase and mortality in maintenance hemodialysis patients. Am J Kidney Dis Off J Natl Kidney Found. 2006 Jul;48(1):59–68.

13. Etter C, Straub Y, Hersberger M, Räz HR, Kistler T, Kiss D, et al. Pregnancy-associated plasma protein-A is an independent short-time predictor of mortality in patients on maintenance haemodialysis. Eur Heart J. 2010 Feb 1;31(3):354–9.

14. Noori N, Kovesdy CP, Dukkipati R, Feroze U, Molnar MZ, Bross R, et al. Racial and ethnic differences in mortality of hemodialysis patients: role of dietary and nutritional status and inflammation. Am J Nephrol. 2011;33(2):157–67.

15. Wang Z, Yu C, Li X hua, Deng B qing. The prognostic value of oxidative stress and inflammation in Chinese hemodialysis patients. Ren Fail. 2017 Jan 1;39(1):54–8.

16. Cho Y, Johnson DW, Vesey DA, Hawley CM, Pascoe EM, Clarke M, et al. Baseline serum interleukin-6 predicts cardiovascular events in incident peritoneal dialysis patients. Perit Dial Int J Int Soc Perit Dial. 2014/04/07 ed. 2015;35(1):35–42.

17. Prelevic V, Antunovic T, Radunovic D, Gligorovic-Barhanovic N, Gledovic B, Ratkovic M, et al. Malnutrition inflammation score (MIS) is stronger predictor of mortality in hemodialysis patients than waist-to-hip ratio (WHR)-4-year follow-up. Int Urol Nephrol. 2021 Jul 13;

18. Lobo JC, Stockler-Pinto MB, Farage NE, Faulin T do ES, Abdalla DSP, Torres JPM, et al. Reduced plasma zinc levels, lipid peroxidation, and inflammation biomarkers levels in hemodialysis patients: implications to cardiovascular mortality. Ren Fail. 2013;35(5):680–5.

19. Is a body mass index of 23 kg/m2 a reliable marker of protein–energy wasting in hemodialysis patients? - ScienceDirect [Internet]. [cited 2021 Nov 17]. Available from: https://www.sciencedirect.com/science/article/pii/S0899900711004710

20. Feldreich T, Nowak C, Fall T, Carlsson AC, Carrero JJ, Ripsweden J, et al. Circulating proteins as predictors of cardiovascular mortality in end-stage renal disease. J Nephrol. 2019 Feb;32(1):111–9.

21. Thang LV, Loc ND, Kien NT, Dung NH, Quyen DBQ, Tuan NM, et al. Interleukin 6 is a better predictor of 5-year cardiovascular mortality than high-sensitivity C-reactive protein in hemodialysis patients using reused low-flux dialyzers. Int Urol Nephrol. 2020 Jun;52(6):1135–42.

22. Hu Z, Zhu F, Zhang N, Zhang C, Pei G, Wang P, et al. Impact of arteriovenous fistula blood flow on serum il-6, cardiovascular events and death: An ambispective cohort analysis of 64 Chinese hemodialysis patients. PloS One. 2017;12(3):e0172490.

23. Bi M, Li B, Li Q. Correlation of hemochromatosis gene mutations and cardiovascular disease in hemodialysis patients. Ann Saudi Med. 2013;33(3):223–8.

24. Lambie M, Chess J, Donovan KL, Kim YL, Do JY, Lee HB, et al. Independent effects of systemic and peritoneal inflammation on peritoneal dialysis survival. J Am Soc Nephrol. 2013;24(12):2071–80.

25. Pecoits-Filho R, Bárány P, Lindholm B, Heimbürger O, Stenvinkel P. Interleukin-6 is an independent predictor of mortality in patients starting dialysis treatment. Nephrol Dial Transplant Off Publ Eur Dial Transpl Assoc - Eur Ren Assoc. 2002 Sep;17(9):1684–8.

26. Holden R, Tuttle A, Burbidge T, Hegadorn C, Grabell J, Pruss C, et al. Quantitative and qualitative changes of von Willebrand factor and their impact on mortality in patients with end-stage kidney disease. Blood Coagul Fibrinolysis. 2013;24:71Y_726.

27. Kimmel PL, Chawla LS, Amarasinghe A, Peterson RA, Weihs KL, Simmens SJ, et al. Anthropometric measures, cytokines and survival in haemodialysis patients. Nephrol Dial Transplant Off Publ Eur Dial Transpl Assoc - Eur Ren Assoc. 2003 Feb;18(2):326–32.

28. Rao M, Guo D, Perianayagam M, Tighiouart H, Jaber B, Pereira B, et al. Plasma interleukin-6 predicts cardiovascular mortality in hemodialysis patients. Am J Kidney Dis. 2005;45(2):324‐333.

29. Kimmel PL, Phillips TM, Simmens SJ, Peterson RA, Weihs KL, Alleyne S, et al. Immunologic function and survival in hemodialysis patients. Kidney Int. 1998 Jul;54(1):236–44.

30. Rao M, Li L, Tighiouart H, Jaber BL, Pereira BJG, Balakrishnan VS, et al. Plasma adiponectin levels and clinical outcomes among haemodialysis patients. Nephrol Dial Transplant Off Publ Eur Dial Transpl Assoc - Eur Ren Assoc. 2008/03/11 ed. 2008 Aug;23(8):2619–28.

31. Löwbeer C, Stenvinkel P, Pecoits-Filho R, Heimbürger O, Lindholm B, Gustafsson SA, et al. Elevated cardiac troponin T in predialysis patients is associated with inflammation and predicts mortality. J Intern Med. 2003 Feb;253(2):153–60.

32. Yu Z, Lambie M, Chess J, Williams A, Do JY, Topley N, et al. Peritoneal Protein Clearance Is a Function of Local Inflammation and Membrane Area Whereas Systemic Inflammation and Comorbidity Predict Survival of Incident Peritoneal Dialysis Patients. Front Physiol. 2019 Feb 18;10:105–105.

33. Hung SC, Hsu TW, Lin YP, Tarng DC. Decoy receptor 3, a novel inflammatory marker, and mortality in hemodialysis patients. Clin J Am Soc Nephrol CJASN. 2012 Aug;7(8):1257–65.

34. Panichi V, Maggiore U, Taccola D, Migliori M, Rizza GM, Consani C, et al. Interleukin-6 is a stronger predictor of total and cardiovascular mortality than C-reactive protein in haemodialysis patients. Nephrol Dial Transplant Off Publ Eur Dial Transpl Assoc - Eur Ren Assoc. 2004 May;19(5):1154–60.

35. Wetmore JB, Lovett DH, Hung AM, Cook-Wiens G, Mahnken JD, Sen S, et al. Associations of interleukin-6, C-reactive protein and serum amyloid A with mortality in haemodialysis patients. Nephrol Carlton Vic. 2008 Oct;13(7):593–600.

36. Tsirpanlis G, Boufidou F, Zoga M, Triantafyllis G, Fatourou A, Stamatelou K, et al. Low cholesterol along with inflammation predicts morbidity and mortality in hemodialysis patients. Hemodial Int Int Symp Home Hemodial. 2009 Apr;13(2):197–204.

37. Lorenz G, Schmalenberg M, Kemmner S, Haller B, Steubl D, Pham D, et al. Mortality prediction in stable hemodialysis patients is refined by YKL-40, a 40-kDa glycoprotein associated with inflammation. Kidney Int. 2018 Jan;93(1):221–30.

38. Kato A, Takita T, Furuhashi M, Maruyama Y, Hishida A. Association between seroprevalence of anti-chlamydial antibodies and long-term cardiovascular mortality in chronic hemodialysis patients. Atherosclerosis. 2006 Sep;188(1):120–5.

39. Bologa RM, Levine DM, Parker TS, Cheigh JS, Serur D, Stenzel KH, et al. Interleukin-6 predicts hypoalbuminemia, hypocholesterolemia, and mortality in hemodialysis patients. Am J Kidney Dis Off J Natl Kidney Found. 1998 Jul;32(1):107–14.

40. Honda H, Qureshi AR, Heimbürger O, Barany P, Wang K, Pecoits-Filho R, et al. Serum albumin, C-reactive protein, interleukin 6, and fetuin a as predictors of malnutrition, cardiovascular disease, and mortality in patients with ESRD. Am J Kidney Dis Off J Natl Kidney Found. 2006 Jan;47(1):139–48.

41. Pachaly MA, do Nascimento MM, Suliman ME, Hayashi SY, Riella MC, Manfro RC, et al. Interleukin-6 is a better predictor of mortality as compared to C-reactive protein, homocysteine, pentosidine and advanced oxidation protein products in hemodialysis patients. Blood Purif. 2008;26(2):204–10.

42. Beberashvili I, Sinuani I, Azar A, Kadoshi H, Shapiro G, Feldman L, et al. Decreased IGF-1 levels potentiate association of inflammation with all-cause and cardiovascular mortality in prevalent hemodialysis patients. Growth Horm IGF Res Off J Growth Horm Res Soc Int IGF Res Soc. 2013 Dec;23(6):209–14.

43. Beberashvili I, Sinuani I, Azar A, Kadoshi H, Shapiro G, Feldman L, et al. Increased basal nitric oxide amplifies the association of inflammation with all-cause and cardiovascular mortality in prevalent hemodialysis patients. Int Urol Nephrol. 2013 Dec;45(6):1703–13.

44. Dai L, Watanabe M, Qureshi AR, Mukai H, Machowska A, Heimbürger O, et al. Serum 8-hydroxydeoxyguanosine, a marker of oxidative DNA damage, is associated with mortality independent of inflammation in chronic kidney disease. Eur J Intern Med. 2019 Oct;68:60–5.

45. Panichi V, Rosati A, Bigazzi R, Paoletti S, Mantuano E, Beati S, et al. Anaemia and resistance to erythropoiesis-stimulating agents as prognostic factors in haemodialysis patients: results from the RISCAVID study. Nephrol Dial Transplant Off Publ Eur Dial Transpl Assoc - Eur Ren Assoc. 2011 Aug;26(8):2641–8.

46. Han SH, Choi HY, Kim DK, Moon SJ, Lee JE, Yoo TH, et al. Elevated cardiac troponin T predicts cardiovascular events in asymptomatic continuous ambulatory peritoneal dialysis patients without a history of cardiovascular disease. Am J Nephrol. 2009;29(2):129–35.

47. Wang AYM, Lam CWK, Chan IHS, Wang M, Lui SF, Sanderson JE. Long-term mortality and cardiovascular risk stratification of peritoneal dialysis patients using a combination of inflammation and calcification markers. Nephrol Dial Transplant Off Publ Eur Dial Transpl Assoc - Eur Ren Assoc. 2009 Dec;24(12):3826–33.

48. Beberashvili I, Sinuani I, Azar A, Kadoshi H, Shapiro G, Feldman L, et al. Low Serum Concentration of Obestatin as a Predictor of Mortality in Maintenance Hemodialysis Patients. BioMed Res Int. 2013 Sep 12;2013:e796586.

49. Carrero JJ, Stenvinkel P, Fellström B, Qureshi AR, Lamb K, Heimbürger O, et al. Telomere attrition is associated with inflammation, low fetuin-A levels and high mortality in prevalent haemodialysis patients. J Intern Med. 2008 Mar;263(3):302–12.

50. Hung C.-Y., Chen Y.-A., Chou C.-C., Yang C.-S. Nutritional and inflammatory markers in the prediction of mortality in Chinese hemodialysis patients. Nephron - Clin Pract. 2005 May;100(1).

51. Hasuike Y, Nonoguchi H, Ito K, Naka M, Kitamura R, Nanami M, et al. Interleukin-6 is a predictor of mortality in stable hemodialysis patients. Am J Nephrol. 2009;30(4):389–98.

52. Liu YL, Liu JH, Wang IK, Ju SW, Yu TM, Chen IR, et al. Association of inflammatory cytokines with mortality in peritoneal dialysis patients. BioMedicine. 7(1):1.

53. Sun J, Axelsson J, Machowska A, Heimbürger O, Bárány P, Lindholm B, et al. Biomarkers of Cardiovascular Disease and Mortality Risk in Patients with Advanced CKD. Clin J Am Soc Nephrol CJASN. 2016 Jul 7;11(7):1163–72.

54. Panichi V, Rizza GM, Paoletti S, Bigazzi R, Aloisi M, Barsotti G, et al. Chronic inflammation and mortality in haemodialysis: effect of different renal replacement therapies. Results from the RISCAVID study. Nephrol Dial Transplant Off Publ Eur Dial Transpl Assoc - Eur Ren Assoc. 2008 Jul;23(7):2337–43.

55. Tripepi G, Mallamaci F, Zoccali C. Inflammation markers, adhesion molecules, and all-cause and cardiovascular mortality in patients with ESRD: searching for the best risk marker by multivariate modeling. J Am Soc Nephrol JASN. 2005 Mar;16 Suppl 1:S83-88.

56. Badiou S, Cristol JP, Jaussent I, Terrier N, Morena M, Maurice F, et al. Fine-tuning of the prediction of mortality in hemodialysis patients by use of cytokine proteomic determination. Clin J Am Soc Nephrol CJASN. 2008 Mar;3(2):423–30.

57. Kalantar-Zadeh K, Kopple JD, Humphreys MH, Block G. Comparing outcome predictability of markers of malnutrition-inflammation complex syndrome in haemodialysis patients. Nephrol Dial Transplant Off Publ Eur Dial Transpl Assoc - Eur Ren Assoc. 2004 Jun;19(6):1507–19.

58. Muzasti RA, Suhardjono D, Purwanto B, Sembiring RJ. Fetuin-A Thr256Ser gene polymorphism as a mortality predictor in patients with chronic kidney disease on maintenance haemodialysis in Medan, Indonesia. Med Glas Off Publ Med Assoc Zenica-Doboj Cant Bosnia Herzeg. 2020 Feb 1;17(1):22–9.

59. Gergei I., Kalsch T., Marz W., Kramer B.K., Kalsch A.-I. Platelet and monocyte activity markers and mortality in patients with end-stage renal disease. Clin Lab. 2020;66(3):439–47.

60. Meuwese CL, Snaedal S, Halbesma N, Stenvinkel P, Dekker FW, Qureshi AR, et al. Trimestral variations of C-reactive protein, interleukin-6 and tumour necrosis factor-α are similarly associated with survival in haemodialysis patients. Nephrol Dial Transplant. 2011 Apr 1;26(4):1313–8.

61. Hayden JA, van der Windt DA, Cartwright JL, Côté P, Bombardier C. Assessing bias in studies of prognostic factors. Ann Intern Med. 2013 Feb 19;158(4):280–6.

**Supplementary material (2): Author judgment and support for quality assessment of the risk of bias:**

1. Section A: Studies which reported the association between IL-6 with all-cause mortality per 1 pg/ml

Title: An intradialytic increase in serum interleukin-6 levels is associated with an increased mortality in hemodialysis patients

Lichtenberg 2015

| Domain | Author’s Judgment | Support for the judgment |
| --- | --- | --- |
| 1. Study Participation (SP) | Unclear | MT: Doesn't specify recruitment period and dates. Does outline clearly inclusion / exclusion, key characteristics of population (albeit in two separate groups). Obaida Date of recruitment is not reported which might represent bias in the selection, definition for the duration of active infection is not reported (week/ month). The presence of 50% were diabetic also might represent that the study sample has more comorbidity. There is a difference in the description of the characteristics between the table and main body of text. |
| 2. Study Attrition | Low | The figure 2 shows that all the patients had been followed up during the 3 years. |
| 3. Prognostic Factor (PF) Measurement | Low | OI The method of measuring IL-6 is well described including the degree lowest detectable level and the name of the used kite. There was no missing data for the measurement of IL-6 (Figure 2). Matt All criteria met, apart from blinding - which isn't in essential criteria for low risk. |
| 4. Outcome Measurement | Low | OI The outcome was clearly considered as all-cause mortality.  Matt Clearly delineates outcomes (including causes of death) and follow up period. |
| 5. Study Confounding | High | Comorbidities were accounted in the study design but none of them included in the survival model. Age has been included in the survival model, however, mean age of the sample population was reported in two different values. |
| 6. Statistical Analysis and Reporting | Low | OI Figures 2 shows that there was no missing data in measuring the prognostic factor IL-6. All the sufficient information (number and name of adjusted factors, effect size, confidence interval, p-value, length of follow up, univariate analysis, number of events or/death, the name of analysis model, the strategy for model building, forward stepwise analysis ) were reported. Two survival models were reported. The model which included three variables has been selected for extraction because the other one is overfitted because it included seven covariates with only 29 events. |

Title: Delta-He: A novel marker of inflammation predicting mortality and ESA response in peritoneal dialysis patients

Danielson 2014

| Domain | Author’s Judgment | Support for the judgment |
| --- | --- | --- |
| 1. Study Participation (SP) | Unclear | OI: There is about more than 50 % participation from eligible patients. The participants are represented for the key characteristics baseline age, sex, and diabetes. However, the inclusion and exclusion criteria were not clear and the authors have not exclude patients with infection, HIV, malignancy. Matt No exclusion, 50% (not < 50%) participation. |
| 2. Study Attrition | Unclear | OI: The drop rate is not reported and we can not judge the attrition rate. Matt There is no reporting of why patients dropped out, and no description of characteristics of those dropping out. |
| 3. Prognostic Factor (PF) Measurement | Low | OI: There was only two missing value for laboratory measurement and they were excluded. Matt All criteria met, apart from blinding / imputation, which are not essential unless described. |
| 4. Outcome Measurement | Low | MT: All-cause mortality, deaths identified during follow up period. Obaida All-cause mortality was the primary outcome and it is not expected to be biased. |
| 5. Study Confounding | High | There are three survival models. Although the first model does not include comorbidity, it has been selected for extraction because the other model is overfitted as it included four variables for only 24 events. However, this make the model at high risk of bias in cofounder domain. |
| 6. Statistical Analysis and Reporting | Unclear for univariable analysis | Continuous value for the effect size and confidence interval were not reported. |
|  | High for multivariable analysis | OI: Continuous value for the effect size and confidence interval were not reported. There is misuse of the concept multivariate model which is a common statistical mistake. In addition, there is overfitting of the model by including four covariates and having only 24 death. So, the model with only three covariates has been selected for extraction. The significant effect of IL-6 has been lost in the model with four covariates after adjustment for the subjective global assessment. Matt No explicit reporting of IL-6 multivariable HR + 95%CI in tabular / text form. |

Title: Inflammation and asymmetric dimethylarginine for predicting death and cardiovascular events in ESRD patients

Tripepi 2011

| Domain | Author’s Judgment | Support for the judgment |
| --- | --- | --- |
| 1. Study Participation (SP) | Unclear | There more details about the inclusion and exclusion criteria is needed including the duration of infection. The study sample represent the population of interest and the baseline is well described, However, there is no information about the place of recruitment, number of center and date of recruitment. |
| 2. Study Attrition | Low | Obaida During the long time follow up 13 years, only 6 % is lost to follow up. There is no description about the reason of lost to follow up, and no details about any difference, however, this unlikely to represent bias. |
| 3. Prognostic Factor (PF) Measurement | Unclear | There is definition of IL-6 and clear description about the method in measuring IL-6, however, the lack of reporting about missing patients samples and the imputation method make the judgment is not clear. |
| 4. Outcome Measurement | Low for all-cause mortality  High for cardiovascular mortality | MT: It is unclear, by mixing CV fatal and non-fatal up, what proportion are deaths. It would have been clearer to just say CV events. Additionally, family recall was involved in determining patient outcomes (for both CV and all-cause mortality). Obaida There is a clear description that five physician reported the cause of death and follow up by telephone when the patients dies out the hospital. |
| 5. Study Confounding | Unclear | Age and diabetic and CV comorbidities were included in the study design and analysis. However, there is no information about the percentage of missing data for cofounders. |
| 6. Statistical Analysis and Reporting | Unclear | OI: They report number and name of adjusted factors, effect size, confidence interval, p-value, length of follow up, number of events or/death and the name of the analysis model. However, the risk of bias is not clear because there is no information about imputation for missing data, univariate analysis, and the strategy in choosing the covariates and building the model. Matt No univariable HR. Not overfitted. |

Title: Comorbidity and Acute Clinical Events as Determinants of C-Reactive Protein Variation in Hemodialysis Patients: Implications for Patient Survival

Snaedal 2009

| Domain | Author’s Judgment | Support for the judgment |
| --- | --- | --- |
| 1. Study Participation (SP) | Low | The sampling study is well described, about 87% from eligible population participate. In addition, the study sample is representing of the population in comorbidity, age, sex. It does not give a summary statistic for IL-6 clearly. However, from figure 3, B it can be seen that IL-6 IOR between 5 and 20. Infectious condition was one of exclusion criteria. |
| 2. Study Attrition | Low | Drop rate is 3%. Almost all the patients were followed up to survival outcome and there is complete description about the reason of lost to follow up. |
| 3. Prognostic Factor (PF) Measurement | Low | They do not report any difference in the measurement between the patients. Routine laboratory measurement was used. Low level of missing data. |
| 4. Outcome Measurement | Low | OI: All-cause mortality was the primary outcome and it is not expected to be biased. Matt Weekly interaction with study participants - likely to get accurate information regarding outcome data. |
| 5. Study Confounding | Low | Age and comorbidities were included in the study design and in the analysis. The percentage of missing data was only for some cofounder smoking and access catheter and it was low. |
| 6. Statistical Analysis and Reporting | Unclear | The univariate analysis is not reported and the effect size of other covariates is not reported. The strategy in building the model and choosing covariates is not explained. It is not clear whether the adjustment for comorbidity was as Davies index in continuous value or as categorical groups. |

Title: IL-6 Levels, Nutritional Status, and Mortality in Prevalent Hemodialysis Patients

Beberashvilli 2011

| Domain | Author’s Judgment | Support for the judgment |
| --- | --- | --- |
| 1. Study Participation (SP) | Unclear | Unclear selection bias might arise from the fact that date of recruitment is not mentioned, we don’t know how many agree to participate. |
| 2. Study Attrition | Low | They have described that they have 24 censored in the analysis due to transplantation or transfer to other haemodialysis centres or peritoneal dialysis. It can be seen from table 3 that they know they know the final outcome for all the patients. |
| 3. Prognostic Factor (PF) Measurement | Unclear | They use the same ELISA with all measurements. No clear description about missing data for measuring the prognostic factor IL-6. |
| 4. Outcome Measurement | Low | Outcome is mortality is no way to misclassify it. No issues - all clearly identified. |
| 5. Study Confounding | Unclear | Age and diabetic and CV comorbidities were included in the study design and analysis. However, there is no information about the percentage of missing data for cofounders. |
| 6. Statistical Analysis and Reporting | High | This is a description that analysis is Cox proportional hazard and IL 6 has been used as a continuous value by the change in 1 pg/ml with reporting P-value, 95% CI, Hazard ratio. However, there is overfitting of the model by including eight covariates and having only 35 events. Furthermore, the univariate analysis is not reported. There is misuse of the concept multivariate model which is a common statistical mistake. In addition, there is no report about missing data and imputation method. |

Title: Plasma Gelsolin and Its Association with Mortality and Hospitalization in Chronic Hemodialysis Patients

Gama 2017

| Domain | Author’s Judgment | Support for the judgment |
| --- | --- | --- |
| 1. Study Participation (SP) | Unclear | There is a description about the sample frame, place of recruitment, and inclusion and exclusion criteria, however, the exclusion does not include the infection status, acute infection, HIV, and auto-immune disease which might affect the prognostic value for IL-6 on all- cause mortality. In addition, the participation rate is not reported. |
| 2. Study Attrition | Low | Drop rate is not exactly clear, however, it is less than 20 % for all-cause mortality. |
| 3. Prognostic Factor (PF) Measurement | Unclear | OI: The method of measuring IL-6 was with the Quantikine ELISA kit (R&D, Minneapolis, Minn, USA) at Spectra Clinical Research (Rockleigh, N.J., USA). However, there is no description about missing blood sample. In addition, it is not clear the type of blood samples, serum or plasma, which was used to measure il-6.  Matt Although not explicitly stated, there is no reason to believe significant proportions of participants did not have IL-6 measurement. The only issue here is whether it is plasma or serum, which is not stated. Therefore unclear. |
| 4. Outcome Measurement | Low | All-cause mortality was the primary outcome and it is not expected to be biased Matt All described correctly - all cause mortality outcome, recorded on monthly intervals from EHR. |
| 5. Study Confounding | High | Obaida Age were included in the study design and analysis. However, comorbidities were accounted in the study design but none of them included in the survival model. In addition to that, there is no information about the percentage of missing data for cofounders.  MT: The issues here which swing towards high bias is the lack of clarity regarding measurement of confounding characteristics, linked with the lack of clarity regarding study population. |
| 6. Statistical Analysis and Reporting | Unclear for univariable analysis | The included number of patients in the model is not reported. No information about the missing data and imputation. There is misuse of the concept multivariate model which is a common statistical mistake. |
|  | High for multivariable analysis | The included number of patients in the model is not reported. No information about the missing data and imputation. There is an overfitting of the model by including 6 variables for 29 events. There is misuse of the concept multivariate model which is a common statistical mistake. Matt Cox, IL-6 increments described, clear method outlined, HR uni + multi reported with CI's, appropriate for design of study. However, the model is overfitted quite spectacularly! |

Title: Serum levels of anti-alpha Galactosyl antibodies predict survival and peritoneal dialysis-related enteric peritonitis rates in patients undergoing renal replacement therapy.

Fontan 2006

| Domain | Author’s Judgment | Support for the judgment |
| --- | --- | --- |
| 1. Study Participation (SP) | Low | The name and the number of centres and countries are not provided clearly. However, it can be expected that there is only one centre in Spain depending on the authors’ affiliation. The participation rate was more than 50%. The study sample represent the population of interest in key characteristics age, sex, Charlson comorbidities despite the fact that there is a higher percentage of diabetes than registries which might reflect some bias. |
| 2. Study Attrition | Low | There was early loss up about 80 patients, however, the reasons is well described and this not expected to rise a bias as the main reason was changing the centre to a closer one. For the long follow up 5 patients out from 133 lost to follow up. |
| 3. Prognostic Factor (PF) Measurement | Unclear | The value 0 in quartile range represent an error in reporting the paper or in the measurement. There is no report about the status of missing data of IL-6 and no information about the imputation. |
| 4. Outcome Measurement | Low | All-cause mortality was the primary outcome and it is not expected to be biased.  Matt Detailed table of final outcomes in study highlighted. |
| 5. Study Confounding | High | Comorbidities were included in the study design and analysis. However, age were accounted in the study design but not included in the survival model. In addition to that, there is no information about the percentage of missing data for cofounders. |
| 6. Statistical Analysis and Reporting | Unclear | Univariate cox survival model is not reported. In addition, the strategy in selecting the covariates is not clear. It is not clear if they put 11 covariates in one model which means the model is overfitted, or if they put it one by one then delete the non-significant covariates. Additionally, the number of missing variables and the way of imputation were not reported. There is misuse of the concept of multivariate model which is a common statistical mistake. |

Title: Hepatocyte growth factor as a long-term predictor for total and cardiovascular mortality in patients on peritoneal dialysis

Janda 2013

| Domain | Author’s Judgment | Support for the judgment |
| --- | --- | --- |
| 1. Study Participation (SP) | Unclear | There is no clear information about comorbidity. In addition, place and date of recruitment, number and name of centre, and inclusion and exclusion criteria were not reported. |
| 2. Study Attrition | Low | The drop rate is 20%. The reason for loss to follow up is the renal transplantation. |
| 3. Prognostic Factor (PF) Measurement | Unclear | There is no information about the missing of IL-6 measurement and the imputation. |
| 4. Outcome Measurement | Low | All-cause mortality was the primary outcome and it is not expected to be biased. |
| 5. Study Confounding | High | Age and comorbidities were included in the study design but not in the analysis. Only univariate analysis for IL-6 was done. In addition to that, there is no information about the percentage of missing data for cofounders and the percentage of comorbidities were not reported. |
| 6. Statistical Analysis and Reporting | Unclear | There is no multivariable model which include IL-6 is including in the analysis. In addition to that, the percentage of missing data is not reported. |

Title: Could depression be a new branch of MIA syndrome?

Ogrizovic 2009

| Domain | Author’s Judgment | Support for the judgment |
| --- | --- | --- |
| 1. Study Participation (SP) | Unclear | Source population is not stated, numbers of eligible patients in centre not given, so unclear what participation rate was. No information about the prevalence of comorbidity in the study sample. There is no clear information about the country, however, it highly that the study was conducted in one country depending on the affiliation of the authors. It is noted that there is a difference centres in the affiliation. |
| 2. Study Attrition | Low | Attrition rate 7%. Reason for withdrawal from cohort not specified. |
| 3. Prognostic Factor (PF) Measurement | Unclear | No information about missing data. |
| 4. Outcome Measurement | Low | All-cause mortality was the primary outcome and it is not expected to be biased. |
| 5. Study Confounding | High | Age and comorbidities were included in the study design but not in the analysis. Only univariate analysis for IL-6 was done. In addition to that, there is no information about the percentage of missing data for cofounders. |
| 6. Statistical Analysis and Reporting | Unclear | Matt No multivariable estimate of IL-6 HR, so potentially unclear biased estimate. However, final multivariable survival analysis (MVSA) assessing BDI score appropriately designed, well fitted although stepwise method used. Likely, logarithmic transformation of IL-6 for linear model and not for survival model.  Obaida: Multivariable analysis was not conducted and not reported for IL-6. There is not report about missing data. They use logarithmic IL-6 for groups’ comparisons, however, I think it is not clear if they use logarithmic or continuous scale of IL-6 in the survival model.  Mark: I don’t think it is certain as the coefficient looks like it is for pg/ml but they do state they log transformed for comparisons. Technically the Cox model is not a comparison, so it is quite plausible that they included it as pg/ml and this is what I suspect they did. Personally it would seem reasonable to me to include it in the meta-analysis as pg/ml as I think this is far more likely what they have done but I accept that it is not possible to be 100% sure that this is correct. |

1. Section B: Studies which reported the association between IL-6 with all-cause mortality per 10 pg/ml:

Title: Low triiodothyronine and survival in end-stage renal disease

Zoccali 2006

| Domain | Author’s Judgment | Support for the judgment |
| --- | --- | --- |
| 1. Study Participation (SP) | Low | OI: The name and the number of centres are provided clearly. Despite the study sample represent the population of interest in key characteristics age, sex, and comorbidities. Despite, excluding the cardio-circulatory congestion patients might lead to bias, this is not expected because only eighteen patients were excluded and the participation rate was more than 50%. Mat: Observational cohort clearly identified from two Italian centres. ESKD on HD. 200 patients. Baseline characteristics described including age, sex, diabetes and vintage. Exclusion criteria clearly identified. |
| 2. Study Attrition | Unclear | OI: There is a clear and complete description about the method in recording the cardiovascular events and all-cause mortality, however, there is no mention about the drop rate. Mat No efforts are made to assess study attrition - therefore it is unclear. |
| 3. Prognostic Factor (PF) Measurement | Unclear | OI: There is no clear description about the number of missing values. Mat Validated method for serum IL6 reported elsewhere. Unclear to what extent there is missing data for IL-6, so risk of bias is unclear regarding prognostic factor measurement. |
| 4. Outcome Measurement | Low | OI: All-cause mortality was the primary outcome and it is not expected to be biased. Mat Physician panel followed up patients, including interview of relatives to ascertain follow up status + outcomes if unable to track outcome via hospital notes / system. |
| 5. Study Confounding | Unclear | Age and comorbidities were included in the study design and analysis. There is no information about the percentage of missing data for confounders. |
| 6. Statistical Analysis and Reporting | Unclear | There is a report about the following: number and name of adjusted factors, effect size, confidence interval, p value, length of follow up, the name of analysis. However model missing data percentage and method to hand it are not reported. The mortality events were 102, however, the number of death in the survival model is not clear. There are another model resulted that Il-6 per 10pg/ ml is not associated with survival. HR 1.03, 95% CI (0.82–1.31), P=0.79. The loss of significance might be due to overfitting the model. In addition to that, univariate analysis is only reported for triiodothyronine. The stepwise or backwise method was not reported and the selection of the potential confounders was probably depending on the authors’ clinical knowledge. Matt Strategy method to build final COX model not outlined clearly. |

Title: Objective Score of Nutrition on Dialysis (OSND) as an alternative for the malnutrition-inflammation score in assessment of nutritional risk of haemodialysis patients

Beberashvili 2010

| Domain | Author’s Judgment | Support for the judgment |
| --- | --- | --- |
| 1. Study Participation (SP) | Unclear | The place of sample and the method of recruitment are reported. States 100 eligible population, of which 81 participated which adequate participation rate, about 75%. However, the date of recruitment is not reported. |
| 2. Study Attrition | Low | OI: Despite there is 21 patients who get transplantation or transferred to heamodialysis or other peritoneal dialysis unit. This is not expected to cause attrition bias. Mat Although only 38 patients completed study, the patients who didn't complete the study were accounted for - 22 died, 10 transplanted, 3 changed dialysis modality and 8 transferred to other units (21 were censored in total). There are reasons outlined for these patients being censored. Likely therefore risk of bias is low. This is further supported by the fact that the authors tried to obtain outcome data for all 81 patients. |
| 3. Prognostic Factor (PF) Measurement | Unclear | OI: Enzyme-linked immunosorbent assay (ELISA) kits (R&D System, Minneapolis, MN, USA) was used to measure IL-6 according to the manufacturer’s protocol. The mean minimal detectable dose (mean MDD) for IL-6 was 0.7 pg/mL. However, there is no report about the missing data of IL-6. Mat Plasma IL6 appropriately reported. Unclear how much missing data however. |
| 4. Outcome Measurement | Low | Not a lot of detail is given on how outcomes were collected. However, all-cause mortality was the primary outcome and it is not expected to be biased. |
| 5. Study Confounding | Unclear | Age and comorbidities were included in the study design and analysis. There is no information about the percentage of missing data for confounders. |
| 6. Statistical Analysis and Reporting | High | There is a report about the following: number and name of adjusted factors, effect size, confidence interval, p value, length of follow up, the name of analysis. However model missing data percentage and method to hand it are not reported. The stepwise or backwise method was not reported and the selection of the potential confounders was probably depending on the authors’ clinical knowledge. The mortality events were 22, however, the number of death in the survival model is not clear. There are three model resulted included Il-6 per 10pg/ ml and all of them are overfitting because of including six of five variables. The model with five variables, less number of variables, has been extracted. |

Title: Serum Myeloperoxidase and Mortality in Maintenance Hemodialysis Patients

Kalantar-Zadeh 2006

| Domain | Author’s Judgment | Support for the judgment |
| --- | --- | --- |
| 1. Study Participation (SP) | Unclear | The name and the number of centres are provided clearly. Despite the study sample represent the population of interest in key characteristics age, sex, and comorbidities. The participation rate is more than 50 %. However, there is no any description or explanation about including 256 only out from 356 in Cox proportional survival model. Matt Clear source population, sampling frame and baseline characteristics described. The issue here is participation is only 356 / 1300 patients in the centre. There is mentioned in the discussion "random selection" of patients without knowledge of inflammatory status, yet the method of randomisation is not specified. Unclear therefore whether this selective participation could be hiding selection bias (particularly given methods aren't crystal clear over this). |
| 2. Study Attrition | Unclear | There is no any report about the patients who lost to follow-up. As a result, the drop rate is not clear. Matt There is insufficient data presented to adequately assess attrition bias. 256 (from 356) included in final Cox model. Unclear whether this is due to attrition or missing data. Note 107 endpoints identified. |
| 3. Prognostic Factor (PF) Measurement | Low | IL-6 immunoassay kits based on a solid phase sandwich enzyme-linked immunosorbent assay using recombinant human IL-6 in the General Clinical Research Center Laboratories of Harbor- UCLAMedical Center. Blood samples were available in 356 patients. This could inference that more than 80% has measured IL-6. |
| 4. Outcome Measurement | Low | All-cause mortality was the primary outcome and it is not expected to be biased. MT: Clear outcome identified, expert review to determine outcomes. 107 deaths in the study. |
| 5. Study Confounding | Unclear | Age and comorbidities were included in the study design and analysis. There is no information about the percentage of missing data for confounders. |
| 6. Statistical Analysis and Reporting | Unclear | There is a report about the following: number and name of adjusted factors, effect size, confidence interval, p-value, length of follow-up, and the name of analysis. However model missing data percentage and the method to handle it are not reported. The mortality events were 91 for 356 patients, however, only 256 were included in the survival model and the number of death in the survival model is not clear. There are three models including Il-6 per 10pg/ ml. All of them have similar effect sizes. The model which has age and comorbidities has been selected for extraction. The first model does not have any comorbidities. The other model is overfitted because of including 14 variables in it. The stepwise or backwise method was not reported and the selection of the potential confounders was probably depending on the authors' clinical knowledge. Matt Univariate and multivariable HR reported, specified model building strategy, results reported using recognised format. However, given the final model controls for 13 covariates and with 107 endpoints, could be argued the analysis is underpowered. |

**Supplementary material 3: QUIPS Tool Algorithm for Judgment the Risk of Bias for this review**

QUIPS tool consists from six domains and each one can be ranked to three levels of bias (High, Moderate, Low)(61). Generally, some adjustments have been made before applying

it.

1) First, "Moderate Risk" of bias category has been replaced by "Unclear Risk".

2) Second, the correct answer for the questions is (I do not know/ unknown) in some studies. So, I use dot for it (.).

**Domain 1:**  **To judge the risk of selection bias:**

This domain assessed whether the study sample was representative of the population of interest (population source).

The following changes were made from the original tool:

- The population source question has been combined with sampling frame question because I do not think there is an important difference between them in this review.

So, in this domain there are the following points/issues/questions:

1. The population source, the sampling frame and recruitment are adequately described, possibly including methods to identify the sample, place of recruitment, and period of recruitment.
2. Inclusion and exclusion criteria are adequately described.
3. Report about adequate description of participation in the study by eligible individuals.
4. The baseline study sample is adequately described for key characteristics.

**Summary point:** The study sample represents the population of interest on key characteristics, sufficient to limit potential bias of the observed relationship between the prognostic factor and outcome.

Comments and explanation of the criteria:

- The **population source** is the **place** which the sample was taken, and therefore all members of this **population** should have a chance of being selected for inclusion in the study.
- The **sampling frame** is a list of all the individuals from the population source.
- We consider the key characteristics are age, sex, IL-6 (Mean OR Median), comorbidities (cardiac and diabetics), and time on dialysis. However, many papers do not include all of them. So, we consider age, sex, comorbidities, and any other one is enough.

**Final decision making for domain 1:**

- Unclear risk of bias:
- We made an assignment of unclear risk of bias if any of the following points have been achieved:

1. The source of population or inclusion/ exclusion criteria is not reported.
2. There is not enough information of key characteristics to judge the representation of study sample to the population of interest.
3. Exclusion criteria does not include acute infection.
4. When the duration of infection in the excluded patients might affect the outcome.
5. The participation rate is not reported.

- Low risk of bias:
- We made an assignment of low risk of bias if all of the following points have been achieved::

1. The study sample is representative of population of interest on key characteristics.
2. Inclusion and exclusion criteria are well chosen to avoid any other factors that might affect the prognostic effect of IL-6.
3. The participation rate was more than 50%.

- High risk of bias:
- We made an assignment of high risk of bias if any of the following points have been achieved:

1. The study sample does not represent the population of interest on key characteristics.
2. The participation rate was less than 50%.

**Domain 2:**  **To judge the risk of study attrition bias:**

In this domain there are the following points/issues/questions:

1. Drop rate is adequate <20 %.
2. Attempts to collect information on participants who dropped out of the study are described.
3. Reasons for loss to follow up are described.
4. Participants lost to follow up are adequately described for key characteristics.
5. There are no important differences between key characteristics and outcomes in participants who completed the study and those who did not.

**Summary Point:**
Loss to follow-up is not associated with key characteristics sufficient to limit potential bias to the observed relationship between the prognostic factor and the outcome.

**Key words:** enrol, participants, drop, follow, survivor.

**Final decision making for domain 2:**

- Unclear risk of bias:
- We made an assignment of high risk of bias if any of the following points have been achieved:

1. Drop rate is not reported.
2. There is not enough information to judge the possibility of differences between key characteristics and outcomes in participants who completed the study and those who did not.

- Low risk of bias:
- We made an assignment of low risk of bias if all of the following points have been achieved::

1. Drop rate is adequate < 20 % with or without explanation.
2. >20% with enough explanation that the loss of follow-up is not expected to affect the outcome (complete random missing data). For example, there are no important differences between key characteristics and outcomes in participants who completed the study and those who did not.

- High risk of bias:
- We made an assignment of high risk of bias if any of the following points have been achieved:

1. Drop rate is > 20 % without enough explanation.
2. There are important differences between key characteristics and outcomes in participants who completed the study and those who did not.
3. The reason for missing outcome is likely to affect the outcome (high level of missing data).

**Domain 3:**  **To judge the risk of measurement bias of the prognostic factor (PF):**

In this domain there are the following points/issues/questions:

1. A clear definition or description of the prognostic factors is provided.
2. Method of prognostic factor measurement is adequately valid and reliable to limit misclassification bias.
3. The prognostic factors measured are blinded for outcome measure.
4. Continuous variables/ cut-off points are reported with the unit of measurement.
5. The method and setting of measurement of PF is the same for all study participants.
6. More than 80% of the study sample has completed data for PF variable.

**Summary point:** Prognostic factor is adequately measured in study participants to sufficiently limit potential bias.

Comments:

Most of the studies does not report the blinding stauts for about the outcome when they measure IL-6 level. In my opinoin, I do not think this will affect the measurement bias in this review as the measurement depend on device using ELISA.

**Final decision making for domain 3:**

- Unclear risk of bias:
- We made an assignment of high risk of bias in any of the following points have been achieved:

1. No information about the method of prognostic factor measurement, unit of measurement, or percentage of study participants.

- Low risk of bias:
- We made an assignment of low risk of bias if all the following were met:

1. A clear definition or description of the prognostic factors is provided.
2. The method and setting of measurement of PF is valid and it is the same for all study participants.
3. Unit of the measurement are reported.
4. More than 80% of the study sample has completed data for PF variable.

- High risk of bias:
- We made an assignment of high risk of bias in any of the following points has been meet:

1. The method and setting of measurement of PF or the unit of measurement is different between study participants or it is not valid.
2. Less than 80% of the study sample has completed data for PF variable.

**Domain 4:**  **To judge the risk of measurement bias of the outcome:**

In this domain there are the following points/issues/questions:

1. A clear definition of the outcome is provided.
2. The method of outcome measurement used is valid and reliable to limit misclassification bias.
3. The method and setting of outcome measurement are the same for all study participants.

**Summary point:** Outcome of interest is adequately measured in study participants to sufficiently limit potential bias.

Comment: Most studies and clinical trial in peritoneal dialysis (PD) are not blinding because blinding is something difficult to achive in dialysis.

**Final decision making for domain 4:**

- Unclear risk of bias:
- We made an assignment of high risk of bias in any of the following points has been meet:

1. No definition of the outcome is reported; this point is very important to consider for cardiovascular events.
2. No information about the method of outcome measurement, especially this is very Important when studying cardiovascular events/mortality. For example, cardiovascular mortality might be misleading accounted because most of the reasons are arrhythmia and this might not accurate the real cause because subarachnoid hemorrhage, massive embolic stroke, or aortic dissection might be indistinguishable from a primary arrhythmic event without an autopsy.

- Low risk of bias:
- We made an assignment of low risk of bias if all the following were met:

1. A clear definition or description of the outcome was given (including duration of follow-up).
2. The outcome of interest was measured similarly for all participants (same method and setting).
3. A valid and reliable outcome measure was used (including avoidance of recall methods).

- High risk of bias:
- We made an assignment of high risk of bias in any of the following points has been meet:

1. Different methods were used for participants with different values of the prognostic factor.
2. An unreliable or non-validated outcome measure was used.
3. The outcome measure relied on participant/carer recall.

**Domain 5:**  **To judge the risk of measurement bias of the cofounders:**

In this domain there are the following points/issues/questions:

1. All-important confounders are measured to ensure survival models are not under-fitted.
2. Clear definitions of the important confounders measured are provided.
3. The method and setting of confounding measurement are the same for all study participants.
4. Important potential confounders are accounted for in the study design.
5. Important potential confounders are accounted for in the analysis.

**Summary point:** Important potential confounders are appropriately accounted for, limiting potential bias with respect to the relationship between prognostic factor and outcome.

**Final decision making for domain 5:**

- Unclear risk of bias:

1. No clear definition about cardiac comorbidities.
2. No information about the methods and settings for measuring the adjusted factors.
3. No information about adjusted factors in the analysis model.
4. No information about the percentage of missing data for cofounders.

- Low risk of bias:

We made an assignment of low risk of bias if all the following requirements were met:

1. Where there was multivariable survival analysis a minimum adjudstmenet was made for age and at least one of comorbidties.
2. Key adjustment factors were measured adequately (more than 80 % of participaints).
3. Methods and settings for measurement of adjustment factors were the same for all participants. If there are differnce, they adjusted for the centre effect in the analysis.

- High risk of bias:

We considered there was a likely high risk of bias as a consequence:

1. Where there was multivariable survival analysis, there was no adjudstmenet for age and at none of comorbidties.
2. Key adjustment factors were reported as not measured adequately (less than 80 % of participaints).
3. Different methods or settings for the measurement of adjustment factors between participaints.

**Domain 6:**  **To judge the risk of measurement bias related to the statistical analysis and presentation:**

In this domain there are the following points/issues/questions:

1. There is sufficient presentation of data to assess the adequacy of the analysis.
2. The strategy for model building is appropriate and is based on a conceptual framework or model.
3. The selected statistical model is adequate for the design of the study.
4. There is a description of the association of the prognostic factor and the outcome, including information about the statistical significance.
5. Continuous variables are reported, or cut-off points are used.
6. There is no selective reporting of results.

**Summary point:** The statistical analysis is appropriate for the design of the study, limiting potential for presentation of invalid or spurious results.

**Comments:**

Question 1: imputation for missing data, number and name of adjusted factors, effect size, confidence interval, p value, length of follow up, univariate analysis, number of events or death in the model, the name of analysis model

Question 2: strategy for model building, the method in choosing covariate (backwise, stepwise, or univariate analysis).

Question 3: the name of the model (Cox or linear), the fitting of the model
Question 4: report of IL-6 about effect size, confidence interval, p value

Question 5: Unit of continuous prognotic factor or the cut-off point of categorical prognostic factor

Question 6: Selective reporting is an important issue in prognostic factor reviews because studies commonly report only factors positively associated with outcomes. Notice: We agreed that the absence of reporting univaraite analysis is not considered selective reporting.

**Final decision making for domain 6:**

- Unlcear risk of bias:

1. There was insufficient detail to assess the adequacy, reporting, and strategy of the analysis approach;
2. Univariable analysis is only avaliable for IL-6.

- Low risk of bias

We made an assignment of low risk of bias if all the following requirements were met:

1. There was sufficient presentation of data to assess the adequacy of the analysis ( imputation for missing data, number and name of adjusted factors, effect size, confidence interval, p value, length of follow up, univariate analysis, number of events or death in the model, the name of analysis model).
2. The strategy for model building, the method in choosing covariate (backwise, stepwise, or univariate analysis).
3. The selected statistical model was adequate for the design of the study.
4. There was no selective reporting of results.

- High risk of bias:

We considered there was a likely high risk of bias as a consequence of any of them:

1. The selected statistical model was inadequate.
2. The strategy of model building is not appropraite or there is an overfitting of the model regardess of the reporting about missing data and imputation status.
3. Results were reported selectively, either on the basis of the findings or statistical significance.

**Supplementary material 4: Statistical tables:**

1. **Analysis of the Global Fluid Study by incident and prevalent cohorts:**

|  | **Univariable Model** | | | **Multivariable Model *** | | |
| --- | --- | --- | --- | --- | --- | --- |
|  | **N^a^** | **Hazard Ratio**  **Per 1pg/ml IL-6**  **[95% Confidence Interval]** | **P-value** | **N^a^** | **Hazard Ratio**  **Per 1pg/ml IL6**  **[95% Confidence Interval]** | **P-value** |
| **Incident Cohort** | 550 | 1.04,  [1.02, 1.05], | < 0.001 | 523 | 1.03,  [1.01, 1.04] | < 0.001 |
| **Prevalent Cohort** | 367 | 1.08,  [1.04, 1.11], | < 0.001 | 347 | HR 1.06,  [1.02, 1.1], | < 0.001 |

^a^ Number of patients with complete data

* Included age (per year), comorbidity (Davies score), plasma IL-6 (per 1 pg/ml), renal clearance (per 10 litter/week), albumin (per 1 g/L), duration of PD (per month).

1. **Forest plot for the non-adjusted effect size of IL-6, analysed in continuous scale,for all-cause mortality in dialysis population after excluding Kalantar- Zadeh 2006**


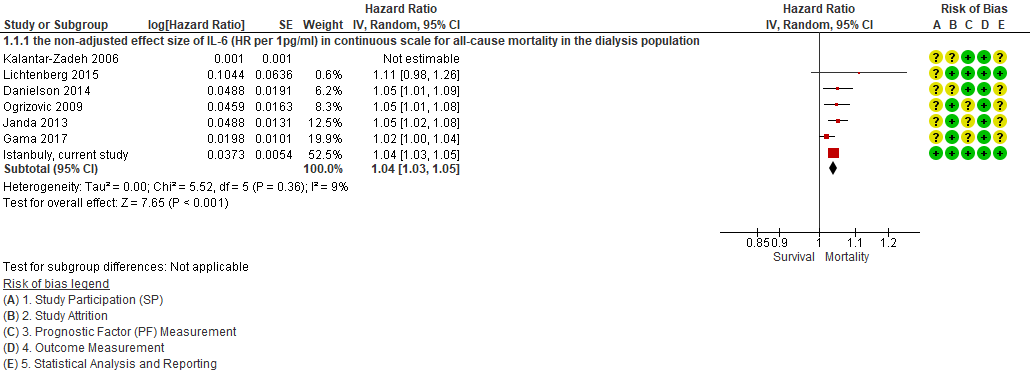


1. **Forest plot for the adjusted effect size of IL-6, analysed in continuous scale, for all-cause mortality in dialysis population after excluding Snaedal 2009 study and Kalantar- Zadeh 2006 study**

**
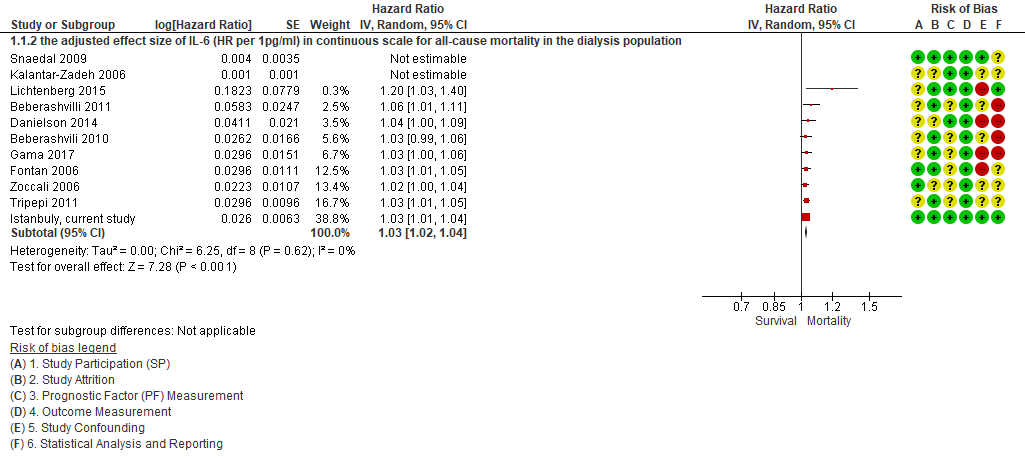
**

**Supplementary material (5): PRISMA 2020 checklists**

- **PRISMA checklist for abstract**

| **Section and Topic** | **Item #** | **Checklist item** | **Reported (Yes/No)** |
| --- | --- | --- | --- |
| **TITLE** | | |  |
| Title | 1 | Identify the report as a systematic review. | Yes |
| **BACKGROUND** | | |  |
| Objectives | 2 | Provide an explicit statement of the main objective(s) or question(s) the review addresses. | Yes |
| **METHODS** | | |  |
| Eligibility criteria | 3 | Specify the inclusion and exclusion criteria for the review. | No |
| Information sources | 4 | Specify the information sources (e.g. databases, registers) used to identify studies and the date when each was last searched. | Yes |
| Risk of bias | 5 | Specify the methods used to assess risk of bias in the included studies. | Yes |
| Synthesis of results | 6 | Specify the methods used to present and synthesise results. | Yes |
| **RESULTS** | | |  |
| Included studies | 7 | Give the total number of included studies and participants and summarise relevant characteristics of studies. | Yes |
| Synthesis of results | 8 | Present results for main outcomes, preferably indicating the number of included studies and participants for each. If meta-analysis was done, report the summary estimate and confidence/credible interval. If comparing groups, indicate the direction of the effect (i.e. which group is favoured). | Yes |
| **DISCUSSION** | | |  |
| Limitations of evidence | 9 | Provide a brief summary of the limitations of the evidence included in the review (e.g. study risk of bias, inconsistency and imprecision). | Yes |
| Interpretation | 10 | Provide a general interpretation of the results and important implications. | Yes |
| **OTHER** | | |  |
| Funding | 11 | Specify the primary source of funding for the review. | No |
| Registration | 12 | Provide the register name and registration number. | Yes |

*From:*  Page MJ, McKenzie JE, Bossuyt PM, Boutron I, Hoffmann TC, Mulrow CD, et al. The PRISMA 2020 statement: an updated guideline for reporting systematic reviews. BMJ 2021;372:n71. doi: 10.1136/bmj.n71

For more information, visit: <http://www.prisma-statement.org/>

- **PRISMA checklist for abstract**

| **Section and Topic** | **Item #** | **Checklist item** | **Location where item is reported** |
| --- | --- | --- | --- |
| **TITLE** | | |  |
| Title | 1 | Identify the report as a systematic review. | page 1 |
| **ABSTRACT** | | |  |
| Abstract | 2 | See the PRISMA 2020 for Abstracts checklist. | Supplementary material 5 page 40-41r |
| **INTRODUCTION** | | |  |
| Rationale | 3 | Describe the rationale for the review in the context of existing knowledge. | Page 3 |
| Objectives | 4 | Provide an explicit statement of the objective(s) or question(s) the review addresses. | Page 3 |
| **METHODS** | | |  |
| Eligibility criteria | 5 | Specify the inclusion and exclusion criteria for the review and how studies were grouped for the syntheses. | Page 4 |
| Information sources | 6 | Specify all databases, registers, websites, organisations, reference lists and other sources searched or consulted to identify studies. Specify the date when each source was last searched or consulted. | Page 4 |
| Search strategy | 7 | Present the full search strategies for all databases, registers and websites, including any filters and limits used. | Page 4 |
| Selection process | 8 | Specify the methods used to decide whether a study met the inclusion criteria of the review, including how many reviewers screened each record and each report retrieved, whether they worked independently, and if applicable, details of automation tools used in the process. | Page 4 |
| Data collection process | 9 | Specify the methods used to collect data from reports, including how many reviewers collected data from each report, whether they worked independently, any processes for obtaining or confirming data from study investigators, and if applicable, details of automation tools used in the process. | Page 5 |
| Data items | 10a | List and define all outcomes for which data were sought. Specify whether all results that were compatible with each outcome domain in each study were sought (e.g. for all measures, time points, analyses), and if not, the methods used to decide which results to collect. | Page 5 |
|  | 10b | List and define all other variables for which data were sought (e.g. participant and intervention characteristics, funding sources). Describe any assumptions made about any missing or unclear information. | Page 5 |
| Study risk of bias assessment | 11 | Specify the methods used to assess risk of bias in the included studies, including details of the tool(s) used, how many reviewers assessed each study and whether they worked independently, and if applicable, details of automation tools used in the process. | Page 5 |
| Effect measures | 12 | Specify for each outcome the effect measure(s) (e.g. risk ratio, mean difference) used in the synthesis or presentation of results. | Page 5 |
| Synthesis methods | 13a | Describe the processes used to decide which studies were eligible for each synthesis (e.g. tabulating the study intervention characteristics and comparing against the planned groups for each synthesis (item #5)). | Page 6 |
|  | 13b | Describe any methods required to prepare the data for presentation or synthesis, such as handling of missing summary statistics, or data conversions. | Page 6 |
|  | 13c | Describe any methods used to tabulate or visually display results of individual studies and syntheses. | Page 6 |
|  | 13d | Describe any methods used to synthesize results and provide a rationale for the choice(s). If meta-analysis was performed, describe the model(s), method(s) to identify the presence and extent of statistical heterogeneity, and software package(s) used. | Page 6 |
|  | 13e | Describe any methods used to explore possible causes of heterogeneity among study results (e.g. subgroup analysis, meta-regression). | Page 6 |
|  | 13f | Describe any sensitivity analyses conducted to assess robustness of the synthesized results. | Page 6 |
| Reporting bias assessment | 14 | Describe any methods used to assess risk of bias due to missing results in a synthesis (arising from reporting biases). | Page 5 |
| Certainty assessment | 15 | Describe any methods used to assess certainty (or confidence) in the body of evidence for an outcome. | Page 6 |
| **RESULTS** | | |  |
| Study selection | 16a | Describe the results of the search and selection process, from the number of records identified in the search to the number of studies included in the review, ideally using a flow diagram. | Pages 9 -10 |
|  | 16b | Cite studies that might appear to meet the inclusion criteria, but which were excluded, and explain why they were excluded. | Page 9 |
| Study characteristics | 17 | Cite each included study and present its characteristics. | Pages 6-7 and supplementary material 1, pages 3-8 |
| Risk of bias in studies | 18 | Present assessments of risk of bias for each included study. | Page 8 and supplementary material 2, pages 14- 28 |
| Results of individual studies | 19 | For all outcomes, present, for each study: (a) summary statistics for each group (where appropriate) and (b) an effect estimate and its precision (e.g. confidence/credible interval), ideally using structured tables or plots. | supplementary material 1, pages 3-10 |
| Results of syntheses | 20a | For each synthesis, briefly summarise the characteristics and risk of bias among contributing studies. | Pages 7 and 23 |
|  | 20b | Present results of all statistical syntheses conducted. If meta-analysis was done, present for each the summary estimate and its precision (e.g. confidence/credible interval) and measures of statistical heterogeneity. If comparing groups, describe the direction of the effect. |  |
|  | 20c | Present results of all investigations of possible causes of heterogeneity among study results. |  |
|  | 20d | Present results of all sensitivity analyses conducted to assess the robustness of the synthesized results. | Page 7 and supplementary material 4, page 38 -39 |
| Reporting biases | 21 | Present assessments of risk of bias due to missing results (arising from reporting biases) for each synthesis assessed. | Pages 8 and 23, and Supplementary material 2, pages 14-28 |
| Certainty of evidence | 22 | Present assessments of certainty (or confidence) in the body of evidence for each outcome assessed. |  |
| **DISCUSSION** | | |  |
| Discussion | 23a | Provide a general interpretation of the results in the context of other evidence. | Page 9 |
|  | 23b | Discuss any limitations of the evidence included in the review. | Pages 9-10– |
|  | 23c | Discuss any limitations of the review processes used. | Pages 9-10 |
|  | 23d | Discuss implications of the results for practice, policy, and future research. | Page 10-11 |
| **OTHER INFORMATION** | | |  |
| Registration and protocol | 24a | Provide registration information for the review, including register name and registration number, or state that the review was not registered. | Page 4 |
|  | 24b | Indicate where the review protocol can be accessed, or state that a protocol was not prepared. | Page 4 |
|  | 24c | Describe and explain any amendments to information provided at registration or in the protocol. | Page 4 |
| Support | 25 | Describe sources of financial or non-financial support for the review, and the role of the funders or sponsors in the review. | Page 13 |
| Competing interests | 26 | Declare any competing interests of review authors. | Page 13 |
| Availability of data, code and other materials | 27 | Report which of the following are publicly available and where they can be found: template data collection forms; data extracted from included studies; data used for all analyses; analytic code; any other materials used in the review. | Page 13 |

*From:*  Page MJ, McKenzie JE, Bossuyt PM, Boutron I, Hoffmann TC, Mulrow CD, et al. The PRISMA 2020 statement: an updated guideline for reporting systematic reviews. BMJ 2021;372:n71. doi: 10.1136/bmj.n71

For more information, visit: <http://www.prisma-statement.org/>


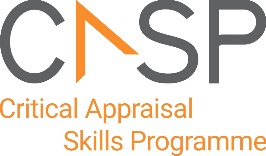


**CASP Checklist:** 10 questions to help you make sense of a **Systematic Review,**

**How to use this appraisal tool:** Three broad issues need to be considered when appraising a systematic review study:


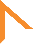
 Are the results of the study valid? (Section A)


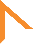
 What are the results? (Section B)


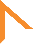
 Will the results help locally? (Section C)

The 10 questions on the following pages are designed to help you think about these issues systematically. The first two questions are screening questions and can be answered quickly. If the answer to both is “yes”, it is worth proceeding with the remaining questions. There is some degree of overlap between the questions, you are asked to record a “yes”, “no” or “can’t tell” to most of the questions. A number of italicised prompts are given after each question. These are designed to remind you why the question is important. Record your reasons for your answers in the spaces provided.

**About:** These checklists were designed to be used as educational pedagogic tools, as part of a workshop setting, therefore we do not suggest a scoring system. The core CASP checklists (randomised controlled trial & systematic review) were based on JAMA 'Users’ guides to the medical literature 1994 (adapted from Guyatt GH, Sackett DL, and Cook DJ), and piloted with health care practitioners.

For each new checklist, a group of experts were assembled to develop and pilot the checklist and the workshop format with which it would be used. Over the years overall adjustments have been made to the format, but a recent survey of checklist users reiterated that the basic format continues to be useful and appropriate.

**Referencing:** we recommend using the Harvard style citation, i.e.: *Critical Appraisal Skills Programme (2018). CASP (insert name of checklist i.e. Systematic Review) Checklist. [online] Available at: URL. Accessed: Date Accessed.*

©CASP this work is licensed under the Creative Commons Attribution – Non-Commercial-Share A like. To view a copy of this license, visit <http://creativecommons.org/licenses/by-nc-sa/3.0/> [www.casp-uk.net](http://www.casp-uk.net/)

**Critical Appraisal Skills Programme (CASP) part of OAP Ltd www.casp-uk.net**

Paper for appraisal and reference:………………………………………………………………………………………….

| Section A: Are the results of the review valid? | | | | |
| --- | --- | --- | --- | --- |
|  | | | | |
| 1. Did the review address a clearly focused question? | Yes | x | | HINT: An issue can be ‘focused’ In terms of  • the population studied  • the intervention given  • the outcome considered |
|  | Can’t Tell |  | |  |
|  | No |  | |  |
|  | | | | |
| Comments:  Yes,  Population is dialysis  The intervention or prognostic factor is IL-6  The outcome is cardiovascular events and mortality and all-cause mortality | | | | |
|  | | | | |
| 1. Did the authors look for the right type of papers? | Yes | x | | HINT: ‘The best sort of studies’ would   - address the review’s question - have an appropriate study design (usually RCTs for papers evaluating interventions) |
|  | Can’t Tell |  | |  |
|  | No |  | |  |
|  | | | | |
| Comments:  Yes, cohort for prognostic studies. | | | | |
|  | | | | |
| Is it worth continuing? | | | | |
|  | | | | |
| 1. Do you think all the important, relevant studies were included? | Yes | x | | HINT: Look for  • which bibliographic databases were used  • follow up from reference lists  • personal contact with experts  • unpublished as well as published studies  • non-English language studies |
|  | Can’t Tell |  | |  |
|  | No |  | |  |
|  |  |  | |  |
| Comments:  Yes, the major database EMBASE, MEDLINE and CENTRAL were searched.  Manual search for the reference lists of the included articles was not conducted  There are two outstanding and global leader in the field supervised this review  The non-published articles was not included  The non-English language studies were translated and screened | | | | |
|  | | | | |
| 4. Did the review’s authors do enough to assess quality of the included studies? | Yes | | x | HINT: The authors need to consider the rigour of the studies they have identified. Lack of rigour may affect the studies’ results (“All that glisters is not gold” Merchant of Venice – Act II Scene 7) |

|  | | | |
| --- | --- | --- | --- |
| Comments:  Yes, QUIPS tool was used.  An algorithm was used to assess the risk of bias. It is provided in the supplementary material, page 25. | | | |
|  | | | |
| 5. If the results of the review have been combined, was it reasonable to do so? | Yes | x | HINT: Consider whether  • results were similar from study to study  • results of all the included studies are clearly displayed  • results of different studies are similar  • reasons for any variations in results are discussed |
|  | Can’t Tell |  |  |
|  | No |  |  |
|  |  |  |  |
|  | | | |
| Comments:  Yes, the results were similar from study to study.  Results were only combined in a meta-analysis if there were more than 3 in the same group using the same reporting method.  reasons for any variations in results are discussed and there is clear descriptions about excluding other studies | | | |
|  | | | |
| Section B: What are the results? | | | |
|  | | | |
| 6. What are the overall results of the review? | | | HINT: Consider  • If you are clear about the review’s ‘bottom line’ results  • what these are (numerically if appropriate)  • how were the results expressed (NNT, odds ratio etc.) |
| Comments:  Results were expressed in hazard ratio and pooled estimate. | | | |
|  | | | |

| 7. How precise are the results? | | | HINT: Look at the confidence intervals, if given |
| --- | --- | --- | --- |
| Comments:  The confidence interval was [1.03, 1.05] for non-adjusted il-6 effect size and [1.01, 1.04] for the adjusted IL-6. | | | |
|  | | | |
| Section C: Will the results help locally? | | | |
|  | | | |
| 8. Can the results be applied to the local population? | Yes |  | HINT: Consider whether   - the patients covered by the review could be sufficiently different to your population to cause concern - your local setting is likely to differ much from that of the review |
|  | Can’t Tell | x |  |
|  | No |  |  |
|  | | | |
| Comments:  Well, this is not systematic review about an intervention. However, the conclusion are applicable to all population. | | | |
|  | | | |
| 9. Were all important outcomes considered? | Yes | x | HINT: Consider whether   - there is other information you would like to have seen |
|  | Can’t Tell |  |  |
|  | No |  |  |
|  | | | |
| Comments:  Yes, all-cause mortality was considered. There were no enough number of studies for cardiovascular events and mortality. | | | |
|  | | | |
| 10. Are the benefits worth the harms and costs? | Yes |  | HINT: Consider   - even if this is not addressed by the review, what do **you** think? |
|  | Can’t Tell | x |  |
|  | No |  |  |
|  | | | |
| Comments:  There no harm by measuring IL-6. | | | |
